# Dynein‐Dependent Endo‐Lysosomal Degradation Drives Lewy Body Disorders Accompanied by Aβ Pathology

**DOI:** 10.1002/advs.202414860

**Published:** 2025-07-18

**Authors:** Linlin Zhou, Yuwei Wang, Yu Liu, Feipeng Zhu, Ge Gao, Chengjie Li, Pu Ai, Jingying Xu, Junxin Wang, Long Guo, Yuting Guan, Virginia Man‐Yee Lee, Jianjun Chen, Jialin Zheng, Qihui Wu

**Affiliations:** ^1^ Shanghai Key Laboratory of Anesthesiology and Brain Functional Modulation Clinical Research Center for Anesthesiology and Perioperative Medicine Translational Research Institute of Brain and Brain‐Like Intelligence Shanghai Fourth People's Hospital Affiliated to Tongji University School of Medicine State Key Laboratory of Cardiology and Medical Innovation Center Shanghai East Hospital School of Medicine Tongji University Shanghai 200092 China; ^2^ State Key Laboratory for Molecular Developmental Biology Institute of Genetics and Developmental Biology Chinese Academy of Sciences Beijing 100101 China; ^3^ Center for Translational Neurodegeneration and Regenerative Therapy Tongji Hospital affiliated to Tongji University School of Medicine Shanghai Frontiers Science Center of Nanocatalytic Medicine The Institute for Biomedical Engineering & Nano Science School of Medicine Tongji University Shanghai 200092 China; ^4^ School of Medicine Tongji University 500 Zhennan Road Shanghai 200331 China; ^5^ Shanghai Frontiers Science Center of Genome Editing and Cell Therapy Shanghai Key Laboratory of Regulatory Biology Institute of Biomedical Sciences and School of Life Sciences East China Normal University Shanghai 200241 China; ^6^ Department of Pathology and Laboratory Medicine Institute on Aging and Center for Neurodegenerative Disease Research Perelman School of Medicine University of Pennsylvania Philadelphia PA 19104 USA

**Keywords:** α‐synuclein, Aβ, dynein, endo‐lysosomal degradation, Lewy body disorder

## Abstract

Dementia with Lewy bodies (DLB) is a significant cause of dementia. However, the limited availability of animal and cellular models that accurately replicate early DLB pathogenesis hampers the understanding of how Aβ plaques influence α‐synuclein (αSyn) pathologies. This study addresses this gap by co‐culturing primary neurons with adult hippocampal brain slices from either wild‐type or Alzheimer's disease (AD) mice containing abundant Aβ plaques and cytokines. Neurons exposed to AD slices showed impaired dynein‐dependent organelle trafficking, reducing endosome–lysosome fusion and causing defective degradation of amyloidogenic αSyn fibrils, thus increasing αSyn inclusions. Notably, an abnormal pre‐accumulation of dynein in AD mice suggests that dysfunctional dynein may serve as a nucleation site for αSyn aggregation upon exposure to pathogenic fibrils. Furthermore, Rab7 activation successfully restored endo‐lysosomal degradation of αSyn fibrils and reduced inclusion formation in mouse models presenting with both Lewy body and Aβ pathologies. These results highlight the dynein‐dependent endo‐lysosomal pathway as a promising therapeutic target for mitigating αSyn‐related pathologies in co‐existing Aβ burden, characteristic of many DLB cases.

## Graphical abstract

This study investigates the impact of Aβ plaques on α‐synuclein (αSyn) pathologies in dementia with Lewy bodies (DLB). Co‐culturing primary neurons with AD mouse brain slices reveals impaired dynein‐dependent organelle trafficking, affecting αSyn degradation. Activating Rab7 restores this process, reducing αSyn inclusions. The findings suggest targeting the dynein‐dependent endo‐lysosomal pathway as a potential therapeutic approach for DLB.

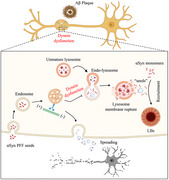

## 1 Introduction

The simultaneous accumulation of α‐synuclein (αSyn), Aβ, and tau is a common feature in neurodegenerative diseases (NDDs). Patients diagnosed with Parkinson's disease with dementia (PDD) and dementia with Lewy bodies (DLB) exhibit neuropathological features, including the presence of Aβ plaques and Lewy body disorders. Approximately 50% of patients with PDD have Aβ plaques in the cortex, which are indicative of pathological changes of Alzheimer's disease (AD).^[^ ^1^ ^]^ These individuals present overlapping clinical symptoms, experience a more rapid decline in cognitive function and motor performance, and have a shortened lifespan compared to other patients without these pathological inclusions.^[^ ^2^, ^3^, ^4^, ^5^, ^6^ ^]^ Pathological examination reveals that Lewy bodies are predominantly distributed throughout the cerebral cortex, particularly in regions such as the hippocampus and amygdala.^[^ ^7^ ^]^ Both DLB and PDD fall under the spectrum of PD disorders rather than being distinct diseases. However, the current lack of cellular or animal models that accurately mimic the disease's progression in its initial stages hinders further evaluation of how Aβ deposition enhances the aggregation and spreading of αSyn pathologies.

αSyn is a soluble presynaptic protein that undergoes post‐transcriptional modifications (PTMs), modulating the formation of phosphorylated aggregates in PD disorders.^[^ ^8^, ^9^ ^]^ Following internalization by cells, αSyn aggregates typically enter the endosomal pathway. One major fate is fusion with lysosomes, leading to degradation, which serves as a vital cellular mechanism to eliminate aggregated proteins and maintain homeostasis.^[^ ^10^ ^]^ However, αSyn aggregates can also be sorted into multi‐vesicular bodies (MVBs). These MVBs may subsequently fuse with the plasma membrane, releasing their contents, including αSyn aggregates, into the extracellular space via exosomes.^[^ ^11^, ^12^, ^13^, ^14^ ^]^ This later process has significant implications for disease progression, as it is believed to contribute to the cell‐to‐cell transmission and spread of αSyn pathology in neurodegenerative diseases.

Most degradative lysosomes are found in the soma and within 25 µm of the dendrite, where bulk degradation of dendritic membrane proteins primarily occurs.^[^ ^15^ ^]^ Retrograde movement of dendritic late endosomes is dependent on Rab7 for fusion with somatic lysosomes, and this movement relies on microtubule‐ and dynein‐dynactin motor complexes. Dynein facilitates autolysosomal transportation toward the soma, where most lysosomes are located, ensuring efficient encounters between autolysosomes and lysosomes.^[^ ^16^ ^]^ As autolysosomes progress from distal to proximal regions, they undergo maturation while becoming increasingly acidic. Both dynein and kinesin play substantial roles in this maturation process and fusion progression.^[^ ^17^ ^]^

However, mis‐sorting and disruption of the lysosomal degradation pathway can accelerate the progression of NDDs. Under neurodegenerative conditions, dysfunctional lysosomes were accumulated within dystrophic neurites in the brains of individuals with AD. Around the plaques, lysosomal acidification, trafficking, and maturation were impaired, resulting in reduced electrical conduction and dysfunction of neural networks.^[^ ^18^, ^19^ ^]^ Moreover, Chu et al. demonstrated the decrease of dynein light chain Tctex type 3 (DYNLT3) in the late stage of PD, and the reduction of DYNLT3 levels was selectively associated with accumulated αSyn inclusions.^[^ ^20^ ^]^ In addition, siRNA‐mediated down‐regulation of dynein resulted in a prolonged half‐life of αSyn and its over‐accumulation in A53T‐overexpressing PC12 cells.^[^ ^21^ ^]^ Moreover, we have previously reported that Aβ deposits dramatically accelerated the pathogenesis and spread of αSyn aggregates in αSyn preformed fibrils (PFFs)‐injected AD mice.^[^ ^22^ ^]^ However, the molecular mechanisms are less well understood.

To explore the potential impact of Aβ plaques on αSyn pathology, αSyn PFFs were incubated with hippocampal neurons co‐cultured with AD brain slices or were administered via stereotaxic injection in transgenic mice with abundant Aβ plaques. Our hypothesis posited that co‐culturing with AD slices or intrahippocampal injection of αSyn PFFs would significantly augment the initiation and propagation of Lewy body disorders accompanied by Aβ pathology.

## 2 Results

### 2.1 Co‐Culture with AD Brain Slices Disrupted Sub‐Cellular Localization of αSyn Fibrillar Seeds

**Figure 1:**
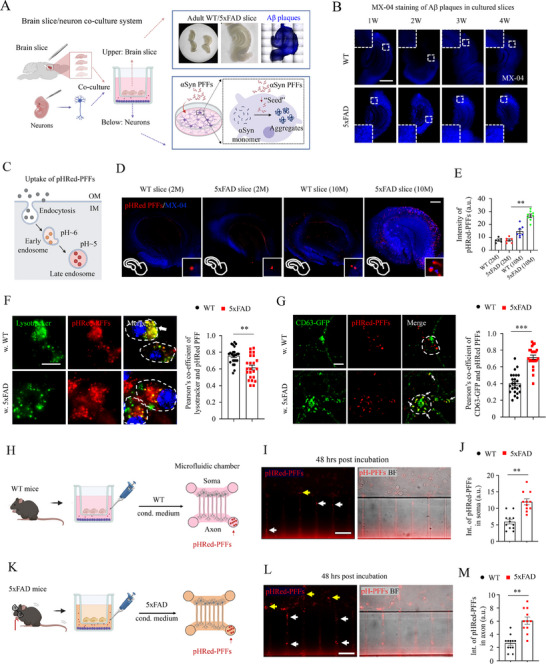
Co‐culture with AD brain slices disrupted subcellular localization of αSyn PFF seeds. A) Schematic procedures depicted a co‐culture system for adult brain slices and hippocampal neurons. Hippocampal slices from 9‐month‐old wild‐type (WT) or 5xFAD mice were cultured on the above surface of membrane inserts, while hippocampal neurons were cultured on the bottom dish of a 6‐well plate. On DIV7, 5 µg of sonicated recombinant human αSyn PFFs or pHRed‐PFFs were diluted to the desired concentration in sterile Dulbecco's PBS (dPBS) and mixed thoroughly in fresh medium by vortexing and added back to the culture medium of each well. 1–7 days later, neurons or slices were collected for live cell observation, immunofluorescent staining, or western blot assays as indicated. B) MX‐04 staining revealed abundant Aβ plaques in the hippocampal brain slices cultured for as long as 4 weeks ex vivo. Scale bar: 200 µm. C) Cartoon illustrates the endocytosis of pHrodo red‐labeled αSyn PFFs (pHRed‐PFFs). pHrodo red is a pH‐sensitive dye that was dimmed in the extracellular neutral solution and can be visualized in acidic organelles when endocytosed.^[^ ^24^ ^]^ D) Representative confocal images of pHRed‐PFFs uptake in the cultured WT and 5xFAD brain slices. Please note, pHRed‐labeled PFF puncta accumulated around MX‐04 positive plaques. Scale bar: 50 µm. E) Statistical quantifications of pHRed‐PFFs. Abbreviation: a.u., arbitrary unit. Student's *t*‐test, **p <0.01. Each dot represents a slice analyzed. F) Representative confocal images and statistical quantifications of lysotracker and αSyn pHRed‐PFFs in neurons co‐cultured with WT or 5x FAD slices. After 5 µg of sonicated pHRed‐PFFs were administered overnight, 5 µMm lysotracker was incubated for 15 min and then captured with confocal microscope. White arrow indicates the colocalization of lysotracker and αSyn pHRed‐PFFs in neurons co‐cultured with WT slices. Right panel: Pearson's co‐efficient of lysotracker and pHRed‐PFFs. Student's *t*‐test, ***p <0.001. Each dot represents a cell analyzed. Scale bar: 10 µm. G) Representative confocal images and statistical quantifications of CD63‐GFP and αSyn pHRed‐PFFs in neurons co‐cultured with WT or 5x FAD slices. CD63‐GFP plasmids were transfected on DIV3 into hippocampal neurons, then these neurons were co‐cultured with brain slices on DIV 7 for at least 3 days. Next, 5 µg of sonicated pHRed‐PFFs were administered overnight and neurons were captured with confocal microscope. White arrow indicates the colocalization of CD63‐GFP and αSyn pHRed‐PFFs in neurons co‐cultured with 5x FAD slices. Right panel: Pearson's co‐efficient of CD63‐GFP and pHRed‐PFFs. Student's *t*‐test, ***p <0.001. Each dot represents a cell analyzed. Scale bar: 10 µm. Abbreviation: w., with. H) Schematic illustration of the co‐culture of WT slice and hippocampal neurons and administration of αSyn pHRed‐PFFs in microfluidic chambers. Neurons were plated and cultured for 7 days for the growth of axons and dendrites in the grooves of microfluidic chambers. The WT slice culture medium was collected and mixed in a 1:1 ratio with fresh hippocampal culture medium to obtain conditioned medium. 5 µg of sonicated pHRed‐PFFs were administered in the axonal chamber and subsequently transported retrogradely into the soma for 48 h. Abbreviation: cond. medium, conditioned medium. I) Representative confocal images of uptake and traffic of αSyn pHRed‐PFFs along axons in WT conditions. Yellow arrows indicate the accumulation of pHRed‐PFFs in soma, white arrows indicate the accumulation of pHRed‐PFFs in axons. Scale bar: 50 µm. J) Statistical quantifications of pHRed‐PFFs in soma. Abbreviations: Int., Intensity; a.u., arbitrary unit. Student's *t*‐test, **p <0.01. Each dot represents a chamber analyzed. K) Schematic illustration of the co‐culture of 5xFAD slice and hippocampal neurons and administration of αSyn pHRed‐PFFs in microfluidic chambers. L) Representative confocal images of uptake and traffic of αSyn pHRed‐PFFs along axons in 5xFAD conditions. Yellow arrows indicate the accumulation of pHRed‐PFFs in soma, white arrows indicate the accumulation of pHRed‐PFFs in axons. Scale bar: 50 µm. M) Statistical quantifications of pHRed‐PFFs in axons. Abbreviations: Int., Intensity; a.u., arbitrary unit. Student's *t*‐test, **p <0.01. Each dot represents a chamber analyzed.

Previously, we established an organotypic brain slice culture system to investigate the impact of neuronal activity on the pathogenesis of αSyn.^[^ ^23^ ^]^ However, how and whether Aβ plaques contribute to αSyn pathogenesis is not fully elucidated. Here, we established an organotypic hippocampal brain slice and primary neuron co‐culture system (**Figure** **1**A). In this system, neurons in the bottom can receive Aβ or inflammatory cytokines that were released from above brain slices, simulating the neuropathological scenario of DLB patients in the brain. Initially, adult hippocampal brain slices were prepared from 9‐month‐old WT or 5xFAD mice and an ELISA assay was performed to characterize the co‐culture system. Strikingly, Aβ plaques can be maintained in brain slices for as long as 4 weeks, as revealed by MX‐04 staining (Figure 1B), along with elevated Aβ42, pro‐inflammatory cytokines, and chemokines in culture medium collected from AD slices than that of WTs (Figure S1, Supporting Information). Importantly, co‐culture with brain slices did not significantly alter the expression levels of neuronal markers, as shown by both western blot analysis and immunofluorescent staining (Figure S2, Supporting Information). Next, we labeled αSyn PFFs with pHrodo red (named as αSyn pHRed‐PFFs), a pH‐sensitive dye that was dimmed in the extracellular neutral solution and can be visualized in acidic organelles when endocytosed (Figure 1C).^[^ ^24^ ^]^ Following our previously established protocol,^[^ ^23^ ^]^ αSyn pHRed‐PFFs were incubated with slice culture medium for 3 days, and only a few pHRed signals were visible in WT slices; however, more αSyn pHRed‐PFFs were taken up into 5xFAD slices and most pHRed signals were accumulated around MX‐04 positive Aβ plaques (Figure 1D,E).

After αSyn PFFs were internalized via endocytosis, the fibrils were trafficked and translocated to endosomes, which can either be delivered into lysosomes for degradation or be sorted into multi‐vesicular bodies (MVBs) for release outside of neurons (Figure S3A, Supporting Information).^[^ ^25^ ^]^ To investigate how internalized αSyn PFFs were processed in the presence of Aβ, we incubated hippocampal neurons with αSyn pHRed‐PFFs for 1 day and found that most αSyn PFFs were not co‐localized with lysotracker in neurons co‐cultured with 5xFAD slices (Figure 1F). Additionally, we transfected hippocampal neurons with CD63‐GFP to label MVBs and then incubated with αSyn pHRed‐PFFs, the results indicated that most αSyn PFFs were co‐localized with MVBs in neurons co‐cultured with 5xFAD slices (Figure 1G), implying a mis‐localization of the fibrils. Consistently, more MVBs were observed in neurons co‐cultured with 5xFAD slices (Figure S3B,C, Supporting Information), indicating an increased biogenesis of MVBs or decreased fusion of MVBs with lysosomes in these neurons.

Then, hippocampal neurons were infected with lentivirus overexpressing CD63‐pHluorin to visualize the synaptic release of MVBs in live cells (Figure S4A,B, Supporting Information). Interestingly, more CD63‐pHluorin fusion events were observed in neurons co‐cultured with 5xFAD slices (Figure S4C, Supporting Information), and more MVBs were released in the full‐fusion‐like (FFL) mode (Figure S4D, Videos S1 and S2, Supporting Information),^[^ ^26^, ^27^, ^28^, ^29^, ^30^ ^]^ implying that more internalized αSyn fibrils can be released in these neurons. Consistently, the increased release of αSyn fibrils was further validated by biochemical and ELISA assays (Figure S5, Supporting Information). These results suggest that in neurons co‐cultured with AD slices, more αSyn PFF seeds were sorted into MVBs and can be released outside of neurons to accelerate the spread of αSyn pathology.

Next, hippocampal neurons were cultured in microfluidic chambers, and αSyn pHRed‐PFFs were incubated in axonal terminals to visualize their retrograde traffic along the grooves (Figure 1H,K). The fibrils were co‐stained with neuronal but not astroglial markers (Figure S6, Supporting Information), indicating that the retrograde transport of αSyn PFF seeds occurs along the neuronal axons rather than through a medium diffusion process. Consistently, increased pHRed signals were observed in both axons and soma of neurons under AD conditions (Figure 1I,J,L,M). Lastly, real‐time live imaging was conducted to observe the transport of lysosomes and αSyn PFF seeds in co‐cultures and microfluidic chambers. As anticipated, axonal trafficking of both lysosomes and αSyn was significantly impaired in neurons under AD conditions. However, in neurons co‐cultured with WT slices, the smaller‐sized lysosomes and αSyn PFF puncta were actively transported (Figures S7–S9, Videos S3–S12, Supporting Information).

Collectively, these findings suggest that co‐culture with AD brain slices disrupts the subcellular localization of αSyn PFF seeds and delays organelle trafficking from distal axons to the soma.

### 2.2 Inhibition of Dynein Disrupted Lysosomal Homeostasis

**Figure 2:**
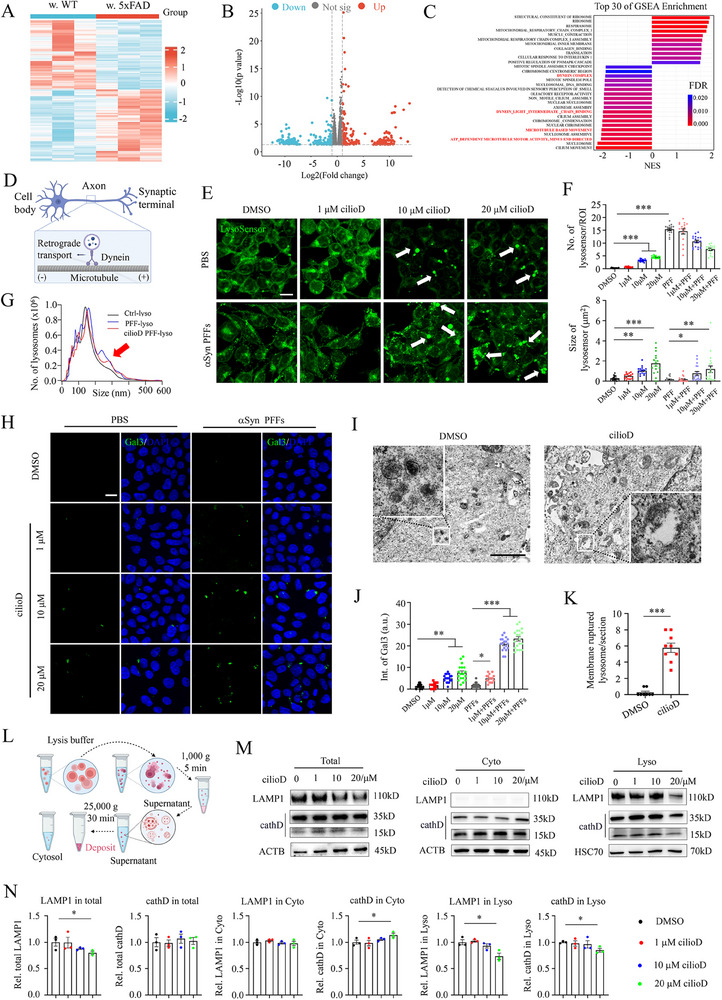
Pharmacological inhibition of dynein disrupted lysosome homeostasis. A,B) Heatmap and volcano plot of bulk RNA sequencing of the hippocampal neurons co‐cultured with WT or 5xFAD slices. C) Top 30 of GSEA enrichment pathways. D) Schematic illustration of dynein‐mediated retrograde transport of cargo from synaptic terminals to soma for lysosomal degradation. E) Representative confocal images of lysosensor in DMSO‐, PFFs‐, or cilioD‐treated SH‐SY5Y cells. White arrows indicate the abnormal acidification or enlarged lysosomes in cilioD and αSyn PFFs‐treated cells. Lysosensor dye is an acidotropic probe that appears to accumulate in acidic organelles as the result of protonation. Scale bar: 20 µm. F) Statistical quantifications of the number and size of lysosensor. One‐way ANOVA test, *p <0.05, **p <0.01, ***p <0.001. Each dot represents a cell analyzed. G. Size distribution of isolated lysosomes with distinct treatments via Nanoparticle Tracking Analysis (NTA) assay. The red arrow indicates the right shift of cilioD and αSyn PFFs treatment‐derived lysosomes. H) Representative confocal images of Gal3 with a serial dose of cilioD and αSyn PFFs treatment. Abbreviation: Gal3, galectin 3. Scale bar: 10 µm. I) Representative transmission electron microscopy (TEM) images of DMSO‐ and cilioD‐treated SH‐SY5Y cells. Zoom‐in graph: cilioD treatment resulted in membrane ruptured and vacuolated lysosomes. Scale bar: 2 µm. J) Statistical quantifications of the intensity of Gal3 puncta of panel H. One‐way ANOVA test, *p <0.05, **p <0.01, ***p <0.001. Each dot represents a cell analyzed. K) Statistical quantifications of the number of ruptured lysosomes per section of panel I. Student's *t*‐test, ***p <0.001. Each dot represents a cell analyzed. L) Schematic illustration of the gradient centrifuge extraction of cytosol and lysosomal fractions. M) Western blot analysis of the expression levels of LAMP1 and cathD in total (left), cytosol (middle), and lysosomal (right) fractions with DMSO or cilioD treatment. Abbreviation: cathD, cathepsin D. lyso, lysosomes. N) Statistical quantifications of the expression levels of LAMP1 and cathD in neurons with DMSO or cilioD treatment. Student's *t*‐test, *p <0.05. Each dot represents a sample analyzed.

To further understand how Aβ plaques disrupted the subcellular localization of αSyn PFFs, we performed bulk RNA sequencing of neurons co‐cultured with WT or 5xFAD slices (**Figure** **2**A). From the analysis, there were 258 upregulated differentially expressed genes (DEGs) and 188 downregulated DEGs, respectively (Figure 2B). From the top 30 of Gene Set Enrichment Analysis (GSEA) pathways, “Dynein complex,” “Dynein light intermediate chain binding,” “Microtubule movement,” and “ATP‐dependent microtubule motor activity” were downregulated in neurons co‐cultured with 5xFAD slices (Figure 2C). However, the expression levels of two dynein complex proteins,Dynein cytoplasmic 1 heavy chain 1 (Dync1h1) and Dynein cytoplasmic 1 light intermediate 1 (Dync1li1), were compensatorily upregulated in AD conditions (Figure S10, Supporting Information). Cytoplasmic dynein 1 is an important microtubule‐based motor in many eukaryotic cells, and dynein–dynactin‐dependent transport machinery is important for lysosome motility, positioning, and tubulation (Figure 2D).^[^ ^31^, ^32^, ^33^ ^]^ Then, ciliobrevin D (cilioD) was used to inhibit the function of dynein in SH‐SY5Y cells, which serves as an extensively used in vitro model for studying dopaminergic neuron‐like behaviors in response to neurotoxins associated with PD development.^[^ ^34^ ^]^ Lysosensor dye was used to indicate the pH of lysosomes (Figure 2E). Unexpectedly, either cilioD or αSyn PFFs administration have dramatically increased the size and number of lysosensor puncta (Figure 2F). Subsequently, lysosomes were enriched from cells that were treated with αSyn PFFs or cilioD. Nanoparticle tracking analysis (NTA) assay revealed a right‐shifted distribution in both αSyn PFFs‐ and cilioD‐treated groups (Figure 2G), indicating an increase in the size of lysosomes.

Next, immunofluorescent staining of galectin‐3 (Gal3) was performed with a serial concentration of cilioD and αSyn PFFs (Figure 2H). The results demonstrated that the intensity of Gal3 puncta exhibited an increase with higher doses of cilioD, which was further augmented in the presence of αSyn PFFs (Figure 2J), suggesting the rupture of the lysosome membrane. Consistent with this phenomenon, more vacuolar lysosomes were observed in the presence of cilioD as revealed by transmission electron microscopy (TEM) assay (Figure 2I,K). Additionally, we performed sequential extraction of cytosol and lysosomal fractions and separated on SDS‐PAGE (Figure 2L). With the increase of cilioD concentrations, there was a decrease in lysosomal membrane protein (LAMP1) and intra‐lysosome enzyme (cathepsin D, cathD) in the lysosomal fraction, while cathD was gradually increased in the cytosol fraction (Figure 2M,N).

Similarly, in contrast to the control group (DMSO), neurons that were treated with cilioD exhibited enlarged lysosomes as revealed by EM assay (Figure S11, Supporting Information), indicating that lysosomes are dysfunctional and have decreased degradative capacity in these neurons. What's more, increasing the concentration of cilioD gradually increased the Gal3 number and enlarged the lysosensor size in both the soma and terminals of primary hippocampal neurons (Figures S12 and S13A,B, Supporting Information). Consistent with previous findings, with higher cilioD concentrations, cathD was released from lysosomes and detected in the cytosol fractions (Figure S13C,D, Supporting Information), indicating lysosomal membrane rupture of the neurons.

Taken together, these findings suggest that co‐culturing with AD slices disrupts dynein‐mediated organelle traffic pathways, whereas pharmacological inhibition of dynein leads to lysosome membrane rupture.

### 2.3 Lysosomal Dysfunction Delayed the Degradation of αSyn PFF Seeds

**Figure 3:**
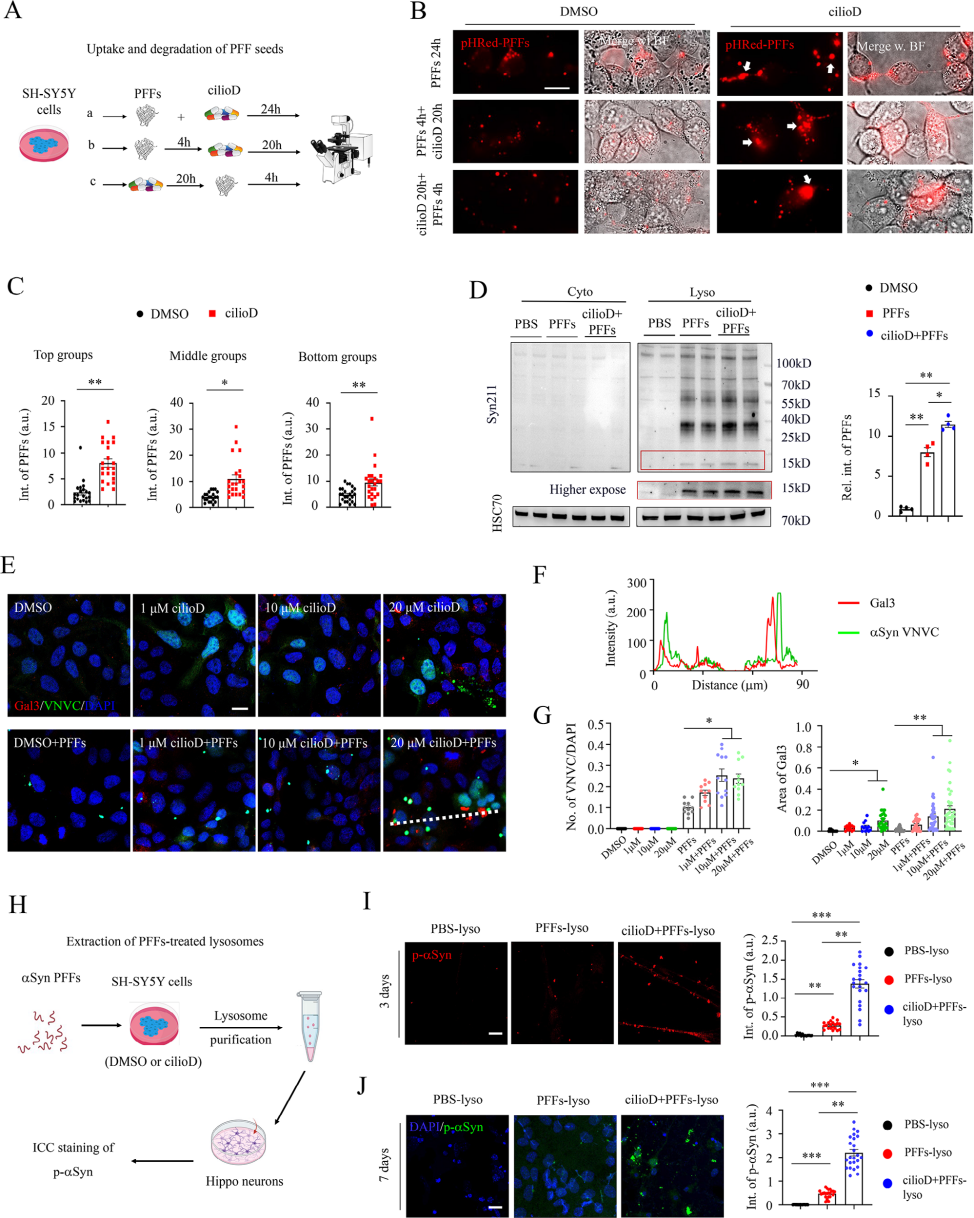
Lysosomal dysfunction delayed the degradation of αSyn PFF seeds. A) Schematic illustration of the αSyn PFF uptake and cilioD administration. B) Representative confocal images of αSyn pHRed‐PFFs with distinct treatments. White arrows indicate the enlarged pHRed puncta. Scale bar: 20 µm. C) Statistical quantifications of intensity of αSyn pHRed‐PFFs in different treatment groups. Student's *t*‐test, *p <0.05, **p <0.01. Each dot represents a cell analyzed. D. Western blot analysis of αSyn PFF seeds in the cytosol and lysosomal fractions with distinct treatments. Syn211 is a human αSyn specific antibody which recognizes an epitope in the C‐terminus region spanning residues 121–125 to detect the undigested PFF seeds. Right panel: Statistical quantifications of undigested αSyn PFFs. One‐way ANOVA test, *p <0.05, **p <0.01. Each dot represents a sample analyzed. E) Representative confocal images of αSyn VNVC and Gal3 with distinct treatments. Scale bar: 10 µm. F) Plot profile of intensity of VNVC and Gal3 along the white‐dashed line in panel (E). G) Statistical quantifications of the number of VNVC (left) and area of Gal3 per DAPI (right). One‐way ANOVA test, *p <0.05, **p <0.01. Each dot represents a cell analyzed. H) Schematic illustration of lysosome purification from αSyn PFFs‐treated cells. SH‐SY5Y cells were incubated with αSyn PFFs, then treated with DMSO or cilioD for one day. The lysosomes were purified and quantified via NTA and BCA assays and used as seeds to induce αSyn pathology in hippocampal neurons. I,J). Representative confocal images and quantifications of p‐αSyn after incubation with isolated lysosomes for 3 days (panel I) and 7 days (panel J) in neurons. Abbreviations: PBS‐lyso, PBS treatment‐derived lysosomes; PFFs‐lyso, αSyn PFFs treatment‐derived lysosomes; cilioD+PFFs‐lyso, cilioD combined αSyn PFFs treatment‐derived lysosomes. One‐way ANOVA test, **p <0.01, ***p <0.001.

Then, we investigated the effects of lysosomal dysfunction on αSyn PFF seeds (**Figure** **3**A,B). Interestingly, regardless of whether cilioD was administered before, after, or concurrently with pHRed‐PFFs, we consistently observed more pHRed signals (Figure 3C), and these fibrils exhibited larger size (Figure 3B, white arrows). This observation suggests that there is an aberrant accumulation of αSyn PFF seeds within dysfunctional lysosomes. Next, SH‐SY5Y cells were incubated with αSyn PFFs, alongside DMSO or cilioD treatment; cytosol and lysosomal fractions were then isolated to investigate the degradation efficiency of internalized fibrils (Figure 3D). From the biochemical analysis, a modest elevation in αSyn fibrillar seeds within the lysosomal fraction was detected following cilioD treatment (Figure 3D), implying more αSyn PFFs remained undigested in lysosomes. Consistently, these lysosomal defects were recapitulated in primary hippocampal neurons by immunofluorescent staining and biochemical assays, respectively (Figure S14, Supporting Information). However, whether these undigested PFFs are functional or not needs further investigation.

Next, we constructed a bimolecular fluorescence complementation (BiFC) probe (the N‐ and C‐terminus of Venus was fused to αSyn, named as αSyn VNVC) that enables visualizing the αSyn aggregation in live cells (Figure S15A, Supporting Information).^[^ ^35^ ^]^ After incubation with PFFs for 3 days, more than 70% of the phosphorylated αSyn (p‐αSyn) was colocalized with VNVC puncta (Figure S15B,C, Supporting Information), indicating the successful report of αSyn aggregation via the VNVC probe. As expected, with the increase in cilioD concentration alone, only a limited number of Gal3 puncta was observed (Figure 3E). However, the presence of both cilioD and αSyn PFFs induced a higher quantity and larger size of VNVC and Gal3 (Figure 3F,G).

Based on these observations, we hypothesized that the undigested PFF seeds within the lysosomes could recruit additional αSyn monomers to form aggregates.^[^ ^24^ ^]^ To test this hypothesis, cells were pre‐treated with PFFs or cilioD for 1 day. Subsequently, the PFF‐containing lysosomes were enriched and utilized as “seeds” to induce αSyn pathology in primary hippocampal neurons (Figure 3H). Strikingly, the cilioD and PFFs co‐treatment‐derived lysosomes induced puncta‐like p‐αSyn signals along neurites as soon as 3 days post incubation (Figure 3I), and continuously recruited more αSyn pathology after incubation for 7 days (Figure 3J). Additionally, DLB patient‐derived brain lysates also induced more Lewy body‐like inclusions and p‐αSyn signals in neurons co‐cultured with 5xFAD slices (Figure S16, Supporting Information).

Overall, these findings provide valuable insights into how lysosomal dysregulation exacerbates the degradation of αSyn PFF seeds, which finally accelerates αSyn pathogenesis.

### 2.4 Knockdown of Dynein Disrupted Lysosome Homeostasis and Induced More αSyn Pathology

**Figure 4:**
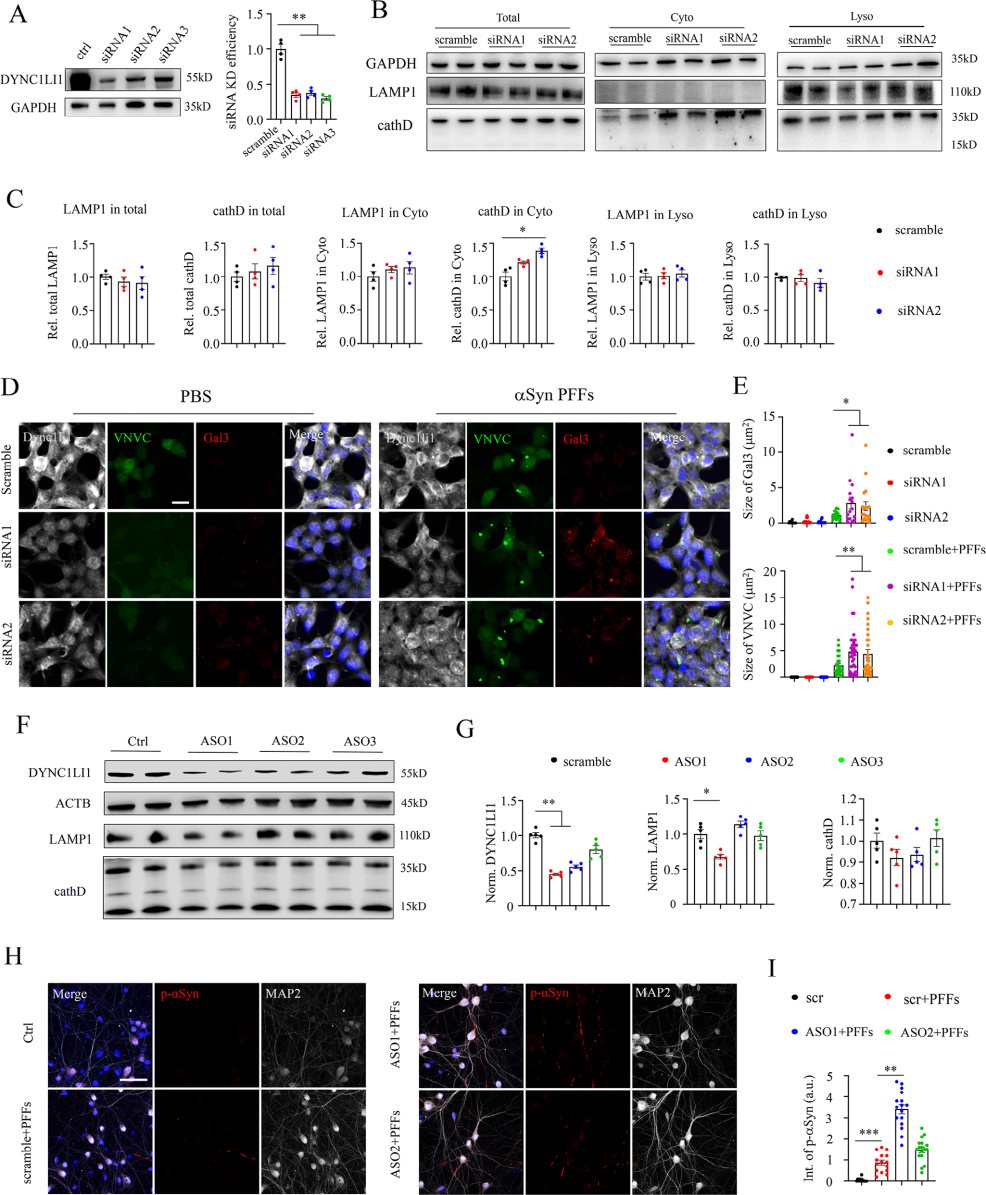
Dynein deficiency disrupted lysosome homeostasis and induced more αSyn pathology. A) Western blot analysis and statistical quantifications of Dync1li1 siRNAs knockdown efficiency. One‐way ANOVA test, **p <0.01. Each dot represents a sample analyzed. B) Western blot analysis of LAMP1 and cathD in total (left), cytosol (middle), and lysosomal (right) fractions after siRNAs‐mediated knockdown of Dync1li1. C) Statistical quantifications of LAMP1 and cathD for panel B. One‐way ANOVA test, *p <0.05. Each dot represents a sample analyzed. D) Representative confocal images of DYNC1LI1, VNVC, and Gal3 after knocking down Dync1li1. Scale bar: 10 µm. E) Statistical quantifications of Gal3 (top) and VNVC (bottom) from panel (D). One‐way ANOVA test, *p <0.05, **p <0.01. Each dot represents a cell analyzed. F) Western blot analysis of DYNC1LI1, LAMP1, and cathD after ASOs‐mediated knockdown of Dync1li1 in hippocampal neurons. G) Statistical quantifications of DYNC1LI1 (left), LAMP1 (middle), and cathD (right) from panel F. One‐way ANOVA test, *p <0.05, **p <0.01. Each dot represents a sample analyzed. H) Representative confocal images of MAP2 and p‐αSyn after knocking down Dync1li1. Scale bar: 50 µm. I) Statistical quantifications of p‐αSyn after knocking down Dync1li1. One‐way ANOVA test, **p <0.01, ***p <0.001. Each dot represents a region of interest (ROI) analyzed.

To further investigate the role of dynein in maintaining lysosomal homeostasis and αSyn pathology, we transfected siRNAs to specifically downregulate dynein expression. Initially, three siRNAs were designed to target the dynein intermediate light chain (DYNC1LI1), which resulted in an efficient knockdown efficiency of approximately 70% (**Figure** **4**A). Next, both cytosol and lysosomal fractions were extracted, and western blot analysis was performed with siRNAs targeting DYNC1LI1. Consistently, knocking down DYNC1LI1 significantly increased cathD levels in the cytosol fraction while mildly reducing LAMP1 abundance in the lysosomal fraction (Figure 4B,C). These findings suggest that dynein deficiency induces lysosomal membrane rupture and subsequently results in lysosomal enzyme leakage.

Furthermore, siRNAs #1 and #2 were transfected into αSyn VNVC cells, followed by incubation with PFFs to induce αSyn pathology (Figure 4D). Interestingly, the results demonstrated that suppressing DYNC1LI1 led to elevated Gal3 signals and increased size of VNVC puncta in the presence of αSyn PFFs (Figure 4D,E). Moreover, we repeated antisense oligonucleotides (ASOs)‐mediated knockdown of DYNC1LI1 in primary hippocampal neurons (Figure 4F). Consistent with pharmacology and siRNA assays (Figures 2M and 4B), the knockdown of DYNC1LI1 dramatically reduced the expression levels of LAMP1 and cathD in neurons (Figure 4G). Additionally, more p‐αSyn pathologies were observed in hippocampal neurons co‐treated with ASOs and PFFs (Figure 4H,I).

Overall, the utilization of siRNAs‐ or ASOs‐mediated knockdown, along with the implementation of a pharmacological inhibitor in this study, has provided additional evidence substantiating the involvement of dynein in the regulation of lysosomal function and its contribution to αSyn pathology. Further investigations are warranted to fully elucidate the underlying mechanisms regarding the molecular interactions between dynein, lysosomes, and αSyn aggregates.

### 2.5 Pre‐Accumulation of Dysfunctional Dynein in AD Mice

**Figure 5:**
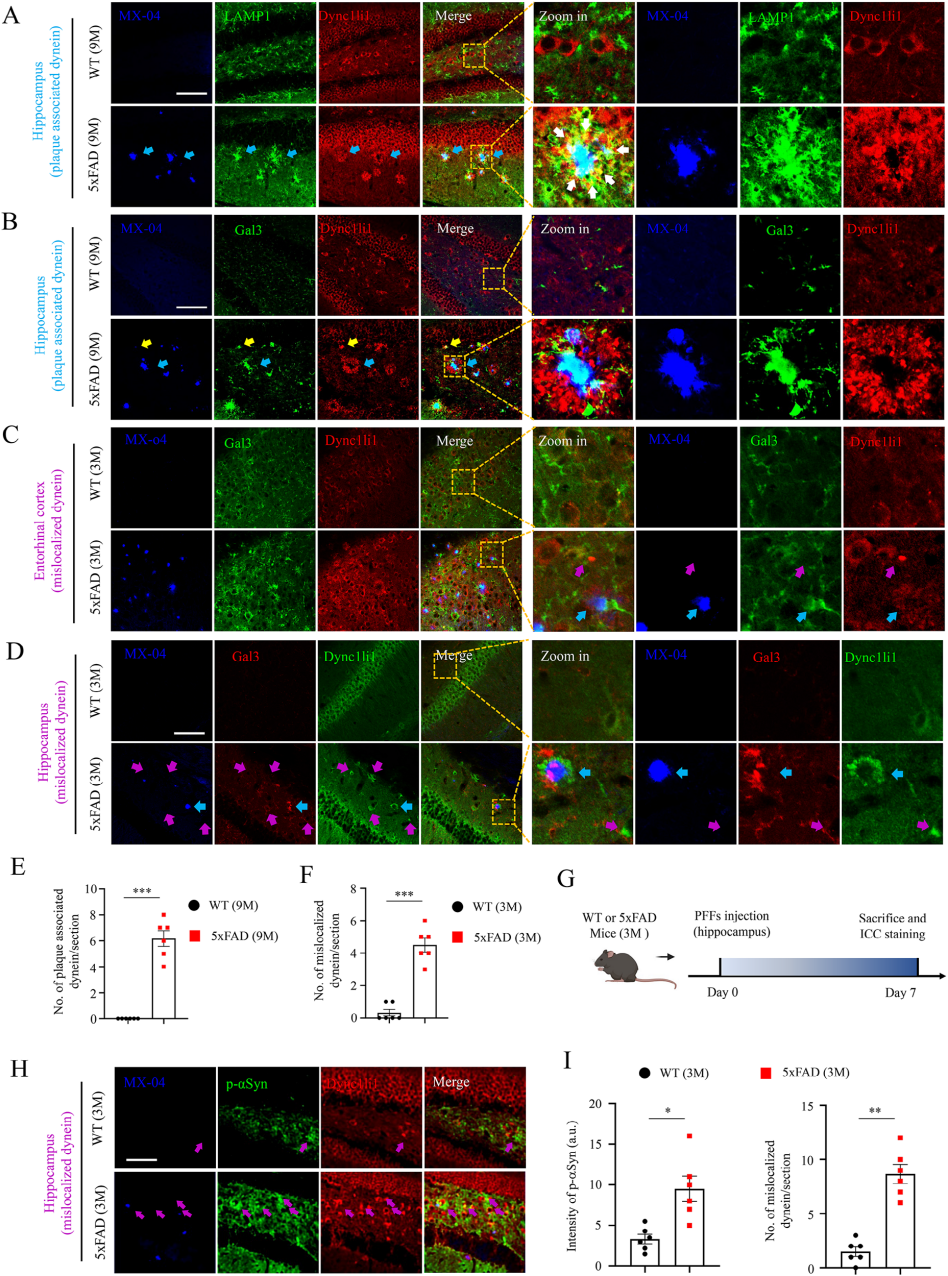
Pre‐accumulation of dysfunctional dynein in AD mice. A) Representative confocal images of MX‐04, LAMP1, and Dync1li1 in 9‐month‐old WT and 5xFAD hippocampal slices. Blue arrows indicate Aβ plaques‐associated dynein. Scale bar: 100 µm. B. Representative confocal images of MX‐04, Gal3, and Dync1li1 in 9‐month‐old WT and 5xFAD hippocampal slices. Blue arrows indicate Aβ plaques‐associated dynein, yellow arrows indicate the abnormal accumulation of Gal3 and dynein without MX‐04 signals. Scale bar: 100 µm. C) Representative confocal images of MX‐04, Gal3, and DYNC1LI1 in 3‐month‐old WT and 5xFAD entorhinal cortical slices. Blue arrows indicate Aβ plaques‐associated dynein, magenta arrows indicate the abnormal accumulation of dynein without Gal3 and MX‐04 signals. Scale bar: 100 µm. D) Representative confocal images of MX‐04, Gal3, and DYNC1LI1 in 3‐month‐old WT and 5xFAD hippocampal slices. Blue arrows indicate Aβ plaques‐associated dynein, magenta arrows indicate the abnormal accumulation of dynein without Gal3 and MX‐04 signals. Scale bar: 100 µm. E) Statistical quantifications of Aβ plaques‐associated dynein in panel (A). Student's *t*‐test, ***p <0.001. Each dot represents a slice analyzed. F) Statistical quantifications of mislocalized dynein in panel D. Student's *t*‐test, ***p <0.001. Each dot represents a slice analyzed. G) Cartoon illustration of the stereotaxic injection of αSyn PFFs in WT and 5xFAD mice, followed by biochemical analysis. H) Representative confocal images of MX‐04, p‐αSyn and DYNC1LI1 in hippocampal slices of 3‐month‐old WT and 5xFAD mice. Magenta arrows indicate the abnormal accumulation of dynein and p‐αSyn. Scale bar: 100 µm. I) Statistical quantifications of intensity of p‐αSyn and mislocalized dynein in panel H). Student's *t*‐test, *p <0.05, **p <0.01. Each dot represents a slice analyzed.

Then, we analyzed the distribution and morphology of dynein and lysosomes in 5xFAD mice. Unexpectedly, like LAMP1 and Gal3, dynein exhibited abnormal accumulation around Aβ plaques (blue arrows, termed as plaque‐associated dynein) in the hippocampus of 9‐month‐old AD mice (**Figure** **5**A–E). Importantly, this dysfunctional dynein was co‐accumulated with LAMP1 and tau around Aβ plaques (Figure S17, white arrows, Supporting Information). Moreover, although the MX‐04 staining was faint, the signals of dynein and Gal3 were already evident (Figure 5B, yellow arrows), implying that dynein and lysosomes could be misfunctioned before the deposition of Aβ plaques. Consistent with this conception, in the 3‐month‐old 5xFAD mice where only limited plaques were present,^[^ ^36^, ^37^ ^]^ we not only observed the co‐distribution of MX‐04, Gal3 and dynein (Figure 5C,D, blue arrows, plaque‐associated dynein), but also noticed abnormal accumulation of dynein alone without Gal3 or MX‐04 signals in both the entorhinal cortex and hippocampus (Figure 5C–F, magenta arrows, termed as mislocalized dynein). Based on these findings, we hypothesized that Aβ‐induced dysfunction of dynein, which disrupted the organelle transport along axons, then impaired lysosomal clearance integrity, and finally accelerated αSyn pathogenesis in the context of Lewy body disorders.

Building on this concept, we inoculated αSyn PFFs into the hippocampus of 3‐month‐old WT or 5xFAD mice and performed biochemical assays (Figure 5G). Following a 7‐day inoculation of αSyn PFFs, an increased presence of p‐αSyn pathologies was observed in the hippocampus of 5xFAD mice (Figure 5H,I), aligning with previous findings from long‐term inoculation studies.^[^ ^22^ ^]^ Most importantly, the p‐αSyn signals were detected surrounding Aβ plaques or dysfunctional dynein in both WT and 5xFAD mice (Figure 5H, magenta arrows), further confirming the hypothesis that the dysfunctional dynein could be the initial site of αSyn pathogenesis.

### 2.6 Aβ Accelerates αSyn Pathogenesis Both In Vitro and In Vivo

**Figure 6:**
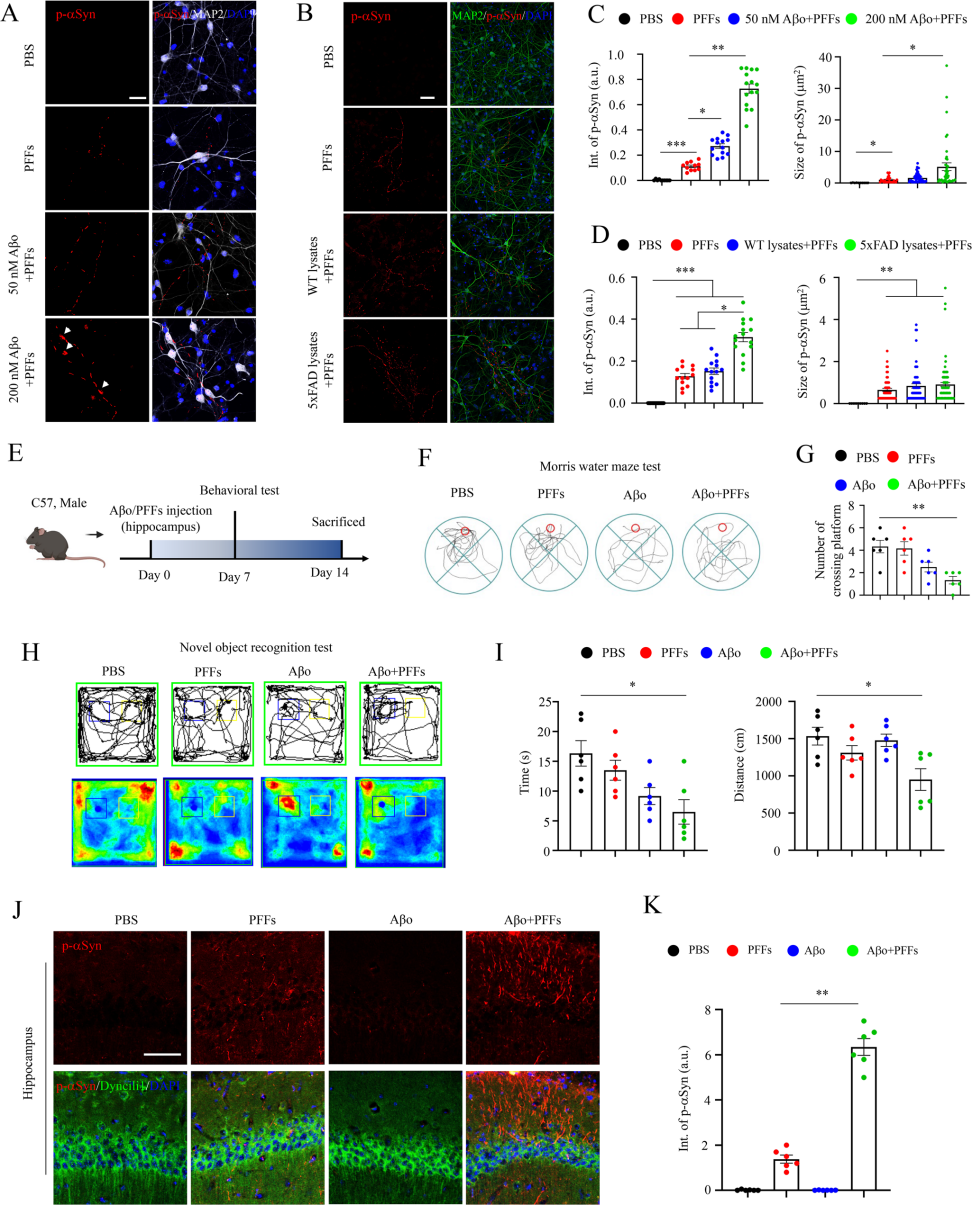
Aβ accelerated p‐αSyn pathogenesis both in vitro and in vivo. A) Representative confocal images of MAP2 and p‐αSyn in hippocampal neurons treated with αSyn PFFs and Aβ oligomers (Aβo). Scale bar: 25 µm. B) Representative confocal images of MAP2 and p‐αSyn in hippocampal neurons treated with αSyn PFFs and brain lysates extracted from 9‐month‐old WT or 5xFAD mice. Scale bar: 25 µm. C) Statistical quantifications of p‐αSyn from panel A. One‐way ANOVA test, *p <0.05, **p <0.01, ***p <0.001. Each dot represents an ROI analyzed. D) Statistical quantifications of p‐αSyn from panel (B). One‐way ANOVA test, *p <0.05, **p <0.01, ***p <0.001. Each dot represents an ROI analyzed. E) Schematic illustration of the stereotaxic injection of αSyn PFFs or Aβo in WT mice, followed by behavior test and biochemical analysis. F) Morris water maze test of mice with distinct treatments. G) Statistical quantifications of total numbers crossed the platform in WT mice with distinct treatments. One‐way ANOVA test, **p <0.01. Each dot represents an animal analyzed. H) Novel object recognition test of mice with distinct treatments. I) Statistical quantifications of time spent on novel object and total distance traveled in WT mice with distinct treatments. One‐way ANOVA test, *p <0.05. Each dot represents an animal analyzed. J) Representative confocal images of DYNC1LI1 and p‐αSyn in hippocampus with distinct treatments. Scale bar: 100 µm. K) Statistical quantifications of p‐αSyn from panel (J). One‐way ANOVA test, **p <0.01. Each dot represents a slice analyzed.

To gain further insights into the underlying mechanisms, we conducted experiments using primary hippocampal neurons. These neurons were treated with either αSyn PFFs or Aβ oligomers (Aβo), and their impacts on lysosome and dynein distribution were investigated. Remarkably, when αSyn PFFs and Aβo were administered together, we observed a significant increase in the co‐localization of LAMP1 and dynein along axons/dendrites (Figure S18, Supporting Information), which is consistent with in vivo studies (Figure 5A). Furthermore, increasing the concentration of Aβo led to elevated p‐αSyn signals when incubated together with αSyn PFFs into hippocampal neurons (**Figure** **6**A–C). What's more, we extracted brain lysates from 9‐month‐old WT or 5xFAD mice and administered together with αSyn PFFs into hippocampal neurons (Figure 6B). Consistently, more p‐αSyn signals were observed in αSyn PFFs and AD lysates co‐treated neurons (Figure 6D). Additionally, this increase in p‐αSyn signals was further confirmed with sarkosyl‐insoluble lysates extracted from AD mouse brains (Figure S19, Supporting Information).

Injection of Aβo into the hippocampus has been applied by several studies to analyze the memory behavior and neuronal loss,^[^ ^38^, ^39^, ^40^, ^41^ ^]^ while injection of αSyn PFFs into WT mice can induce p‐αSyn pathology as soon as 3 days in the synaptic terminals and gradually spreads to the soma.^[^ ^42^ ^]^ Next, both αSyn PFFs and Aβo were injected into the hippocampus of 3‐month‐old WT mice, and behavior tests were performed 7 days later (Figure 6E). Interestingly, αSyn PFFs and Aβo co‐injection resulted in more severe defects in the Morris water maze and novel object recognition tests (Figure 6F–I), while the open field and Y‐maze tests showed no significant changes (Figure S20, Supporting Information). Consistent with these behavior defects, an increased accumulation of αSyn pathologies was observed in the hippocampus co‐injected with αSyn PFFs and Aβo (Figure 6J,K), and these p‐αSyn pathologies were co‐stained with neuronal markers (Figure S21, Supporting Information).

Taken together, our study provides compelling evidence for the detrimental effects of αSyn PFFs and Aβo on neuronal function. The disruption of key cellular processes such as dynein‐mediated transport and lysosomal homeostasis may contribute to the progression of Lewy body disorders. These findings highlight potential targets for therapeutic interventions aimed at restoring intracellular trafficking functions in neurodegenerative diseases involving protein aggregation.

### 2.7 Activation of Rab7 Rescued αSyn Pathology in 5xFAD Mice Injected with αSyn PFFs

**Figure 7:**
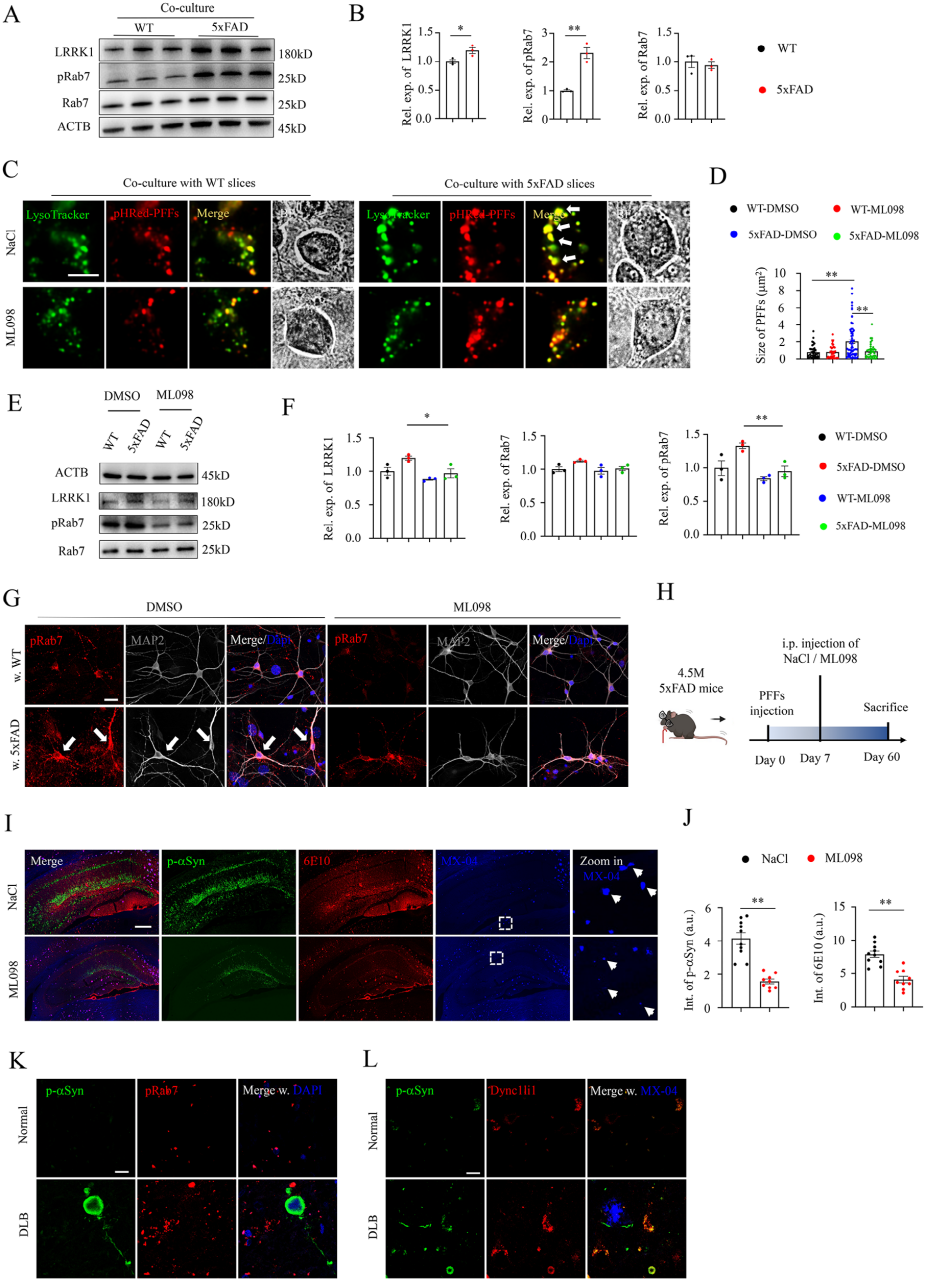
Activation of Rab7 rescued p‐αSyn pathology and Aβ plaques in 5xFAD mice injected with αSyn PFFs. A) Western blot analysis of LRRK1, Rab7, and pRab7 in neurons co‐cultured with WT or 5xFAD slices. B) Statistical quantifications of LRRK1, Rab7, and pRab7. Student's *t*‐test, *p < 0.05, **p < 0.01. Each dot represents a sample analyzed. C) Representative confocal images of lysotracker and αSyn pHRed‐PFFs in neurons co‐cultured with WT (left) or 5xFAD (right) slices with NaCl or Rab7 agonist ML098 treatment. White arrows indicate the enlarged lysosomes in neurons co‐cultured with 5xFAD slices. BF: bright field. Scale bar: 10 µm. D) Statistical quantifications of the size of pHRed‐PFFs from panel C. One‐way ANOVA test, **p < 0.01. Each dot represents a cell analyzed. E) Western blot analysis of LRRK1, pRab7 and Rab7 with distinct treatments. F) Statistical quantifications of LRRK1, pRab7, and Rab7 with distinct treatments. One‐way ANOVA test, *p < 0.05, **p < 0.01. Each dot represents a sample analyzed. G) Representative confocal images of pRab7, Rab7, and MAP2 in hippocampal neurons with distinct treatments. Scale bar: 50 µm. H) Cartoon illustration of the stereotaxic injection of αSyn PFFs in 4.5‐month‐old 5xFAD mice, followed by intraperitoneal injection of NaCl or ML098 for 6 weeks and biochemical analysis. I) Representative confocal images of p‐αSyn, 6E10, and MX‐04 in αSyn PFFs‐injected 5xFAD mice with NaCl or ML098 treatment. White arrows indicate the MX‐04‐labeled Aβ plaques. Scale bar: 200 µm. J) Statistical quantifications of p‐αSyn (left) and 6E10 (right). Student's *t*‐test, **p < 0.01. Each dot represents a slice analyzed. K) Representative confocal images of p‐αSyn and pRab7 in mesencephalon tissues of age‐matched normal and DLB patients. Scale bar: 20 µm. L) Representative confocal images of p‐αSyn, MX‐04, and Dync1li1 in mesencephalon tissues of age‐matched normal and DLB patients. Scale bar: 20 µm.

Leucine‐rich repeat kinase 1 (LRRK1) phosphorylates Rab7 on S72 at the endosomal membrane, which facilitates the dynein‐driven transport of endosomes toward the perinuclear for degradation.^[^ ^43^ ^]^ Then, we investigated the expression levels of LRRK1, Rab7, and phosphorylated Rab7 (p‐Rab7) in neurons co‐cultured with WT or 5xFAD slices (**Figure** **7**A). Unexpectedly, both LRRK1 and p‐Rab7 were paradoxically increased in neurons co‐cultured with 5xFAD slices (Figure 7B). In addition, p‐Rab7 was abnormally aggregated in neurites and perinuclear in neurons co‐cultured with 5xFAD slices (Figure S22, Supporting Information). However, the mRNA transcripts of LRRK1 showed a compensative downregulation in the bulk RNA‐seq dataset (Figure 2B), further highlighting the importance of Rab7 in maintaining the homeostasis of the endo‐lysosomal degradation system.

Next, hippocampal neurons were treated with the Rab7 GTPase activator, ML098, to investigate the degradation capacity of αSyn PFF seeds. Strikingly, in neurons that were co‐cultured with 5xFAD slices, the enlarged lysotracker and pHRed‐PFF puncta were successfully reduced in ML098‐treated neurons (Figure 7C,D), indicating that the Rab7‐dependent endo‐lysosome degradation system is responsible for the clearance of amyloidogenic seeds. Additionally, ML098 treatment also rescued the expression and distribution of p‐Rab7 in hippocampal neurons as revealed by western blot and immunofluorescent staining (Figure 7E–G).

To further investigate the impact of ML098 in mice with Lewy body disorders, αSyn PFFs were inoculated into the hippocampus of 3‐month‐old 5xFAD mice via stereotaxic injection, and vehicle or ML098 was intraperitoneally injected for 6 weeks (Figure 7H). Importantly, administration of ML098 alleviated not only areas of Aβ plaques but also αSyn PFFs‐induced p‐αSyn pathologies in 5xFAD mice (Figure 7I,J). Additionally, the intensity of Aβ plaques‐associated microglia was reduced in the hippocampus and cortex of ML098‐treated mice, while astrocyte levels were only reduced in the cortex (Figure S23, Supporting Information).

Lastly, we investigated the expressions and distributions of p‐αSyn, MX‐04, DYNC1LI1, and p‐Rab7 in mesencephalon tissues of age‐matched normal patients and patients with DLB. Consistent with animal findings, both p‐αSyn and p‐Rab7 showed a dramatic increase in DLB samples, together with elevated aggregation of p‐αSyn and dynein surrounding MX‐04‐labeled plaques (Figure 7K,L). Moreover, the MVB and lysosome fusion‐ and MVB biogenesis‐related genes also showed disrupted expression patterns in human dopamine neurons, together with reduced expression levels of the DYNC1LI1 gene in human SN tissues (Figure S24, Supporting Information).^[^ ^44^, ^45^ ^]^

Collectively, these findings suggest that activation of Rab7 rescued the endo‐lysosome degradation system, which finally mitigated comorbidities in mouse models with Lewy body disorders accompanied by Aβ pathology.

## 3 Discussion

**Figure 8:**
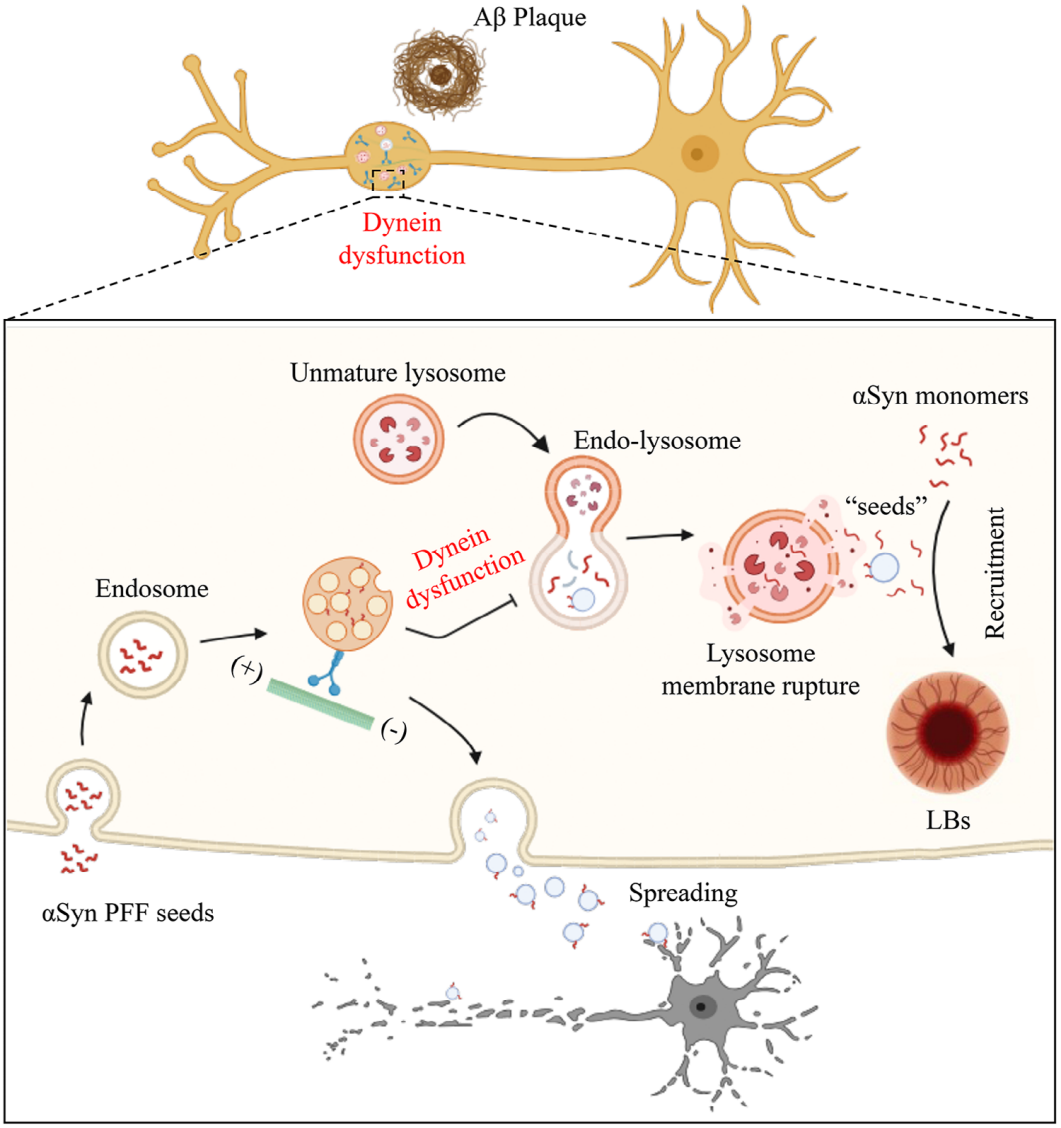
Cartoon illustration depicting dynein‐dependent endo‐lysosomal degradation in Lewy body disorders accompanied by Aβ pathology. Aβ disrupted the function of dynein and delayed intracellular traffic and degradation of αSyn fibrils within lysosomes, which resulted in lysosome membrane rupture. The undigested αSyn fibril “seeds” then recruited more αSyn monomers into Lewy bodies. Moreover, the undigested fibrils can also be released outside of cells via MVBs, which accelerated the spread of pathologies. Abbreviations: N, nuclear; ER, endoplasmic reticulum; GB, Golgi body; LBs, Lewy bodies. Cartoon illustration was created by BioRender and was modified in PowerPoint.

In the present study, we have established a co‐culture system of adult hippocampal brain slices and primary neurons, enabling the investigation of the impact of Aβ and inflammatory cytokines on αSyn pathogenesis. Notably, αSyn PFFs exhibited mis‐localization into MVBs and slower degradation kinetics in neurons co‐cultured with AD brain slices. RNA sequencing revealed a substantial dysfunction of the dynein‐dependent endo‐lysosome degradation pathway. Pharmacological inhibition and genetic deletion of dynein resulted in lysosomal membrane rupture and increased p‐αSyn pathology upon αSyn PFFs incubation. Lastly, activation of Rab7 restored lysosomal degradation of αSyn PFF “seeds” and mitigated αSyn pathologies in an initial‐stage mouse model with Lewy body disorders accompanied by Aβ pathology (**Figure** **8**).

Specifically, the brain slice and neuron co‐culture system established in this study expands the application of organotypic brain slice cultures. We and others have previously cultured brain slices to induce tau, Aβ, and αSyn pathogenesis,^[^ ^23^, ^46^, ^47^, ^48^, ^49^, ^50^, ^51^, ^52^, ^53^ ^]^ as well as screen tau antibodies.^[^ ^54^ ^]^ However, most of the previous studies lacked the use of animal models characterized by abundant Aβ plaques and elevated cytokines within the brain slices, which is essential for accurately mimicking the pathological environment of DLB. Additionally, few studies have explored the application of slice‐neuron co‐culture systems, such as the one developed in the current investigation. This novel approach allows for a more comprehensive understanding of the interactions between Aβ pathology and αSyn aggregation, thus providing a valuable framework for studying the underlying mechanisms of DLB and potential therapeutic interventions. By culturing neurons in the bottom dish with both Aβ and cytokines released from the above slices, αSyn PFFs can be used to induce p‐αSyn pathology that is observed in patients with DLB. However, a previous study injecting αSyn PFFs into 5xFAD mice lacks mechanistic insights into molecular regulators that are involved in this pathological comorbidity process.^[^ ^22^ ^]^

Additionally, dynein‐dependent organelle traffic is significantly disrupted in neurons co‐cultured with AD brain slices. Dynein‐dependent retrograde traffic of autolysosomes is crucial for their fusion with somatic lysosomes, and this fusion process relies on the presence of Rab7 protein.^[^ ^32^, ^55^, ^56^, ^57^ ^]^ Dysfunction of dynein not only hampers the movement of autolysosomes but also profoundly impedes the degradation of amyloidogenic “seeds” within lysosomes, as evidenced by a series of biochemical and live imaging assays. Indeed, pharmacological inhibition or genetic knockdown of dynein replicated the disruptions in lysosome functions and increased αSyn pathologies seen in this study. This finding aligns with two previous studies where reduced levels of DYNLT3 or down‐regulation of dynein led to accumulation of αSyn inclusions or prolonged half‐life of αSyn, respectively.^[^ ^20^, ^21^ ^]^ Furthermore, abnormal distribution patterns were observed for lysosomes within 5xFAD brain slices.^[^ ^18^, ^58^ ^]^ Most importantly, we identified that dynein was found to be accumulated before the formation of Aβ plaques and ruptured lysosomes. These findings suggest that dynein plays a critical role in maintaining proper lysosomal functions and modulating the integrity of the endo‐lysosome degradation system. Additionally, the link between dynein/dynactin dysfunction and tau toxicity has been reported in early studies,^[^ ^59^ ^]^ and the 5xFAD transgenic mouse model also exhibits pathological tau accumulation in their brains.^[^ ^60^ ^]^ These studies further expand the substantial function of dynein in neurodegenerative diseases.

Mechanistically, the transcripts of LRRK1 exhibited a significant reduction as revealed by RNA sequencing analysis of neurons from the AD co‐cultures. However, the protein level of LRRK1 was paradoxically increased through western blot assay, which resulted in the abnormal aggregation of its substrate p‐Rab7 along axons and dendrites and inhibited its proper function. LRRK1 is a kinase that regulates autophagic flux by controlling Rab7 activity in autolysosome formation.^[^ ^61^ ^]^ Deletion of LRRK1 rendered mice susceptible to starvation and disrupted autolysosome formation, as evidenced by the accumulation of enlarged autolysosomes containing undegraded LC3‐II and persistently elevated levels of Rab7‐GTP.^[^ ^61^ ^]^ Malik et al., using mass spectrometry, reported that knockout cells for LRRK1 significantly inhibited phosphorylation of Rab7A at Ser72.^[^ ^62^ ^]^ In our present study, activation of Rab7 with ML098 successfully restored proper localization and degradation of αSyn PFFs seeds within lysosomes and ameliorated p‐αSyn pathology when injecting αSyn PFFs into 5xFAD mice. These findings further validate the crucial role played by the LRRK1‐Rab7‐lysosome signaling pathways in Lewy body disorders.

Lastly, understanding the mechanisms underlying these processes could potentially lead to novel therapeutic strategies aimed at restoring normal lysosomal functions and reducing αSyn pathology in individuals with Lewy body disorders accompanied by Aβ plaques. Further research is warranted to elucidate the precise molecular interactions between Aβ, dynein, lysosomes, and αSyn aggregates, which could provide valuable insights into disease progression and potential targets for intervention.

## 4 Experimental Section

### Animals

All animal procedures were approved by the Tongji University Institutional Animal Care and Use Committee Care and Use of Laboratory Animals. The approval number from the ethics committee is TJBH025211102. Timed‐pregnant C57BL/6 mice or adult 5xFAD mice (Shanghai Model Organisms Center) were used for primary neuronal cultures and in vivo studies, respectively. 3‐month‐old male C57BL/6 mice were used for in vivo Aβo and PFFs injection studies.

### Recombinant αSyn PFFs Purification and In Vitro Fibrillization

Full‐length human αSyn (1‐140) proteins were expressed in BL21 (DE3) RIL cells and purified as previously described,^[^ ^63^ ^]^ and concentrations of endotoxin in the purified monomers were measured to be less than 0.1 EU per µg by the LAL method.^[^ ^64^ ^]^ Fibrillization was conducted by diluting recombinant αSyn to 5 mg mL^−1^ in sterile Dulbecco's PBS (Procell; pH 7.4, without Ca^2+^ or Mg^2+^) followed by incubating this recombinant αSyn at 37 °C with constant agitation at 1000 rpm. for 7 days. Assembled αSyn PFFs were aliquoted and stored at −80 °C.

### Labeling of αSyn PFFs with pHrodo Red

αSyn PFFs were sonicated and labeled with pHrodo Red Microscale Labeling Kit (P35363 or P36600, ThermoFisher Scientific, US) according to manufacturer's instructions. Briefly, transfer 100 µL of a 1 mg mL^−1^ solution of αSyn PFFs (100 µg) to a reaction tube. Add 1/10 volume of 1 m sodium bicarbonate (pH 8.3) and mix by pipetting up and down several times. Add 10 µL DMSO to one vial of pHrodo Red succinimidyl ester. Completely dissolve the contents of the vial by pipetting up and down. Based on the amount of protein you wish to label, determine the amount of reactive dye to use that will give you a dye‐to‐protein molar ratio (MR) of 5–20 moles of dye per mole of protein. Add the appropriate amount of reactive dye to the protein solution in sodium bicarbonate buffer and mix by pipetting up and down several times. Incubate the reaction for 60 min at room temperature, protected from light. Equilibrate a 10 × 300‐mm column with PBS or buffer of choice. The excluded fraction, which corresponds to the first fluorescent band to elute, is the conjugate. For the Catalog # P36600 labeling kit, the labeled proteins can be purified with a microdialysis apparatus for small volumes of proteins (Pierce Chemical Company). Cell lines or primary neurons were treated with 1 µg mL^−1^ of sonicated pHrodo Red‐labeled PFFs for 1–2 days for uptake assays.

### Primary Neuron Cultures and Fibrils Incubation

Primary neuronal cultures were prepared from E14‐E16 C57BL/6 mouse brains obtained from the Shanghai Model Organisms Center. Dissociated hippocampal neurons were plated onto poly‐D‐lysine (Sigma)‐coated coverslips (Biosharp) or dishes at 20 000–100 000 cells cm^−2^ in culture medium (Neurobasal medium (ThermoFisher Scientific, US) supplemented with B27 (ThermoFisher Scientific, US), 2 mMm GlutaMax (ThermoFisher Scientific, US), and 100 U mL^−1^ penicillin/streptomycin (ThermoFisher Scientific, US). Most experiments were performed at 7–21 days in vitro (DIV). αSyn PFFs were diluted in sterile PBS without Ca^2+^/Mg^2+^ and sonicated, then diluted in culture media before being added to cultures.^[^ ^65^ ^]^

### Organotypic Hippocampal Slice and Primary Neuron Co‐Cultures

As we previously described the ex vivo culture of brain slices,^[^ ^23^ ^]^ the hippocampus of 9‐month‐old 5xFAD mice or wild‐type mice were dissected and prepared coronal sections with a thickness of 350 µm using Mcllwain Tissue Chopper (Stoelting, US). These fresh samples were kept in ice‐cold Krebs buffer (in mm): 1.2 NaH_2_PO_4_, 126 NaCl, 2.5 KCl, 1.2 MgCl_2_, 2.5 CaCl_2_, 25 NaHCO_3_, 10 D‐glucose, 10 HEPES, 1% (v/v) penicillin/streptomycin in ultrapure sterile filtered (0.22 µm) H_2_O, pH 7.4. 5–6‐consecutive slices were positioned on Millicell culture inserts (MilliporeSigma, US) for culture in six‐well plates (ThermoFisher Scientific, US) containing 1 mL of sterile slice culture medium. The slices were cultured in the culture medium (100 mL containing: 77.5 mL Neurobasal‐A, 2 mL B27 supplement, 20 mL heat‐inactivated horse serum, 0.5 mL GlutaMAX, 1% (v/v) penicillin/streptomycin) and incubated at 37 °C in a 5% CO_2_ humidified incubator. The brain tissues rapidly attach to the membranes and receive nutrition from the slice culture medium through the membrane via capillary action. Next, pre‐warmed and fresh 20%, 10%, and 1% horse serum and serum‐free brain slice medium were used sequentially for complete fluid exchange with two‐day intervals, followed by continuous incubation with serum‐free brain slice medium. Brain slices were cultured at 37 °C with 5% CO_2_ with a 1:1 mix of fresh and old medium, followed by fluid exchange with 2‐day intervals.

Slices were cultured for a minimum of 7 days before treatment. The membrane inserts containing the brain slices can be placed into a six‐well plate with primary neurons on the bottom layer and co‐cultured by adding 1 mL of serum‐free medium. Exchange half the volume of fresh medium every two days. On DIV7, sonicated recombinant αSyn PFFs or pHrodo‐PFFs were diluted to the desired concentration in sterile Dulbecco's PBS (dPBS) and mixed thoroughly in fresh medium by vortexing and added back to the culture medium of each well. 1–7 days later, neurons or slices were collected for live cell observation, immunofluorescent staining, or western blot assays as indicated.

### Production and Purification of Lentivirus

Briefly, lentiviruses were produced by transfecting the HEK293T cells with the pFUGW backbone vectors expressing CD63‐pHluorin or αSyn A53T and three helper plasmids (pVSVg, RRE, and REV).^[^ ^66^ ^]^ The transfection of plasmids was carried out using the lipofectamine (lipo) method with the ratio at lipo:pFUGW:pVSVg:RRE:REV  =  24:3:1:2:2. 48 or 72 h post transfection, collect the medium and spin at 3 000 g for 5 min, and followed with a 0.45 µm filtration (Millipore). The infectious lentiviruses were collected from the supernatant and further purified by using the lentivirus concentration solution (41101ES50, Yeasen) according to the manufacturer's instructions. The concentrated viruses were resuspended in Ca^2+^ and Mg^2+^‐free PBS and stored at −80 °C until use. Then, WPRE and albumin genes were used as templates for an SYBR green‐based real‐time qPCR method to assess lentiviral copy number. For transfection of hippocampal neurons, lentivirus was added on DIV3 (MOI = 3), the culture medium was fully changed on DIV7 with conditioned medium. Live imaging of hippocampal neurons was taken on DIV8‐10.

### Generation and Culture of αSyn A53T and VNVC Bimolecular Fluorescence Complementation (BiFC) HEK 293T Stable Cell Lines

HEK 293T cells were transfected with αSyn A53T overexpressing lentivirus, or VN‐alpha Synuclein‐pcDNA3.1 and VC‐alpha Synuclein‐pcDNA3.1 (Plasmid #89470, #89471, Addgene) plasmids using Lipofectamine Plus according to the manufacturer's instructions. 48 h post transfection, transfected cells were selected using a selection medium containing 200 µg mL^−1^ of puromycin (Invitrogen) or G418 (Beyotime). The individual colonies were then isolated using cloning discs (Sigma). The overexpression of plasmids was confirmed by western blot and immunofluorescence. All cell lines were cultured in Dulbecco's Modified Eagle Medium (DMEM, high glucose) supplemented with 10% fetal bovine serum and 1% (v/v) penicillin/streptomycin and incubated at 37 °C in a 5% CO_2_ incubator. Maintenance of the αSyn A53T cell line required the addition of 20 ng mL^−1^ puromycin in the medium. For the verification of VNVC aggregation, VNVC HEK 293T stable cells were plated in 24‐well plate, 0.5 µg of sonicated αSyn PFFs were incubated for 3 days and stained with p‐αSyn antibody (Biolegend, 825701). Images were acquired on a confocal fluorescence microscope (Olympus).

### Frozen Section and Staining of Mouse Brains

Fixed brains were sectioned at 30 µm thickness using an FS800 Frozen slicer (RWD). Slices were stored in an antifreeze solution containing 50% PBS, 30% glycerol, and 20% ethanol. Sections were blocked and permeabilized with 5% BSA and 0.5% Triton X‐100 in PBS for 1 h at room temperature. The sections were then treated with primary antibodies diluted in Universal Antibody Diluent Buffer (WB500D, NCM Biotech) at 4 °C overnight. After washing 3 times with PBS, the sections were treated with Alexa488‐, Alexa568‐, or Alexa647‐conjugated secondary antibodies (1:1000, ThermoFisher Scientific, US) for 1 h at room temperature. Nuclei were stained with Hoechst dye by incubation for 15 min at room temperature. After washing, sealed with ProLong Glass Antifade Mountant (P36980, ThermoFisher Scientific, US). Images were acquired on a confocal fluorescence microscope (Olympus). A full list of antibodies and reagents used in this study can be found in Table S1 (Supporting Information).

### Bulk RNA Sequencing

Total RNA was extracted using Trizol reagent (#15596018, ThermoFisher Scientific, US) following the manufacturer's instructions. The quantity and purity of total RNA were analyzed by Bioanalyzer 2100 and RNA 6000 Nano LabChip Kit (Agilent, CA, USA, 5067‐1511). High‐quality RNA samples with an RIN number > 7.0 were used to construct a sequencing library. The RNA libraries were sequenced on the Illumina Novaseq 6000 platform by LC Bio Technology CO.,Ltd (Hangzhou, China). We aligned reads from all samples to the rat or mouse reference genome using HISAT2^[^ ^67^ ^]^ (https://daehwankimlab.github.io/hisat2/, version: hisat 2‐2.0.4). StringTie and ballgown^[^ ^68^ ^]^ were used to estimate the expression levels of all transcripts and perform expression abundance for mRNAs by calculating FPKM (fragment per kilobase of transcript per million mapped reads) value. Differential gene expression analysis was performed using DESeq2 software between two different groups (and edgeR between two samples).^[^ ^69^ ^]^

### Immunofluorescent Staining and Data Analysis

Cells were fixed with 4% PFA for 15 min, washed with PBS for 3 times, then permeabilized and blocked with 3% BSA and 0.25% Triton X‐100 in PBS for 60 min. Cells were incubated with primary antibodies (Table S1, Supporting Information) overnight at 4 °C and the appropriate AlexaFluor (488, 568, 647) conjugated secondary antibodies were used at a dilution of 1:1000. Images were acquired on a confocal fluorescence microscope (Olympus). The analysis of immunofluorescent images followed our previously established protocol.^[^ ^28^ ^]^ Specifically, for colocalization analysis, all intracellular puncta within 1‐µm optical sections were selected and analyzed using the JACoP plugin of McMaster Biophotonics Facility ImageJ software (National Institutes of Health). For the intensity measurements, the background noise signals were subtracted with the “Image‐Adjust‐Threshold” function, and immunofluorescent intensity was calculated with the “Analyze‐Measure” function, the “Mean” value was used to generate the dot plot graphs.

### Isolation and Purification of Lysosomes

After washing 2 times with PBS, collect the cell precipitate by trypsin digestion and centrifugation at 1000 g for 5 min. Add Reagent A, mix well and shake at 4 °C for 10 min. Homogenize with a Dounce homogenizer 30–50 times, and then centrifuge at 2500 g for 10 min, collect the supernatant, and discard the precipitant. The supernatant was further centrifuged at 27 000 g for 30 min, and the cytosol fraction was obtained. PBS or Reagent B was added to the precipitate and blown to mix well, which was the lysosome fraction (Solarbio, EX1230). The size distribution of lysosomes was determined using a nanoparticle tracking analysis (NTA) system (Malvern's NanoSight NS300).

### Brain Lysates Extraction

9‐month‐old WT or 5xFAD brain lysates were prepared by mixing brain tissues with PBS at a ratio of 1: 9. After fully douncing, the samples were centrifuged at 12 000 rpm. for 30 min at 4 °C. The supernatant was used as brain lysates and stored at ‐80 °C. To extract sarkosyl‐insoluble lysates from mouse brain for seeding experiments, hippocampi were dissected from WT or 5xFAD (10 months) mice.^[^ ^70^ ^]^ The tissues were homogenized with 9 volumes of PBS with 0.1% sarkosyl, the homogenates were centrifuged at 10 000 g for 10 min, the low‐spin pellets were discarded, and the supernatants were further centrifuged at 100 000 g for 45 min. The higher‐spin pellets were then washed twice with PBS and finally resuspended in a small volume of PBS. All the PBS used in our experiments were Ca^2+^ and Mg^2+^ free. After quantification of BCA, use them to treat cells at 1 µg mL^−1^.

### Stereotaxic Injection of αSyn PFFs and Aβo

For the in vivo injections, Aβo was used at 0.5 µg per site, αSyn PFFs were used at 5 µg per site, the total volume was 1 µL per site. PBS was used as control.

### Immunoblot Analyses

Briefly, cells were scraped into M‐PER cell lysate buffer (78501, ThermoFisher Scientific, US) containing protease and phosphatase inhibitor on ice. Cell lysates were sonicated and centrifuged at 12 000 rpm. for 10 min. The supernatant was collected and added to 5X SDS‐loading buffer and denatured at 95 °C for 10 min. Samples were separated by SDS‐PAGE and immunoblotting was performed using primary antibodies described in Table S1 (Supporting Information), followed by HRP‐conjugated secondary antibodies (Abmart). Proteins were visualized using ECL (Epizyme) in a Tanon 4600 automatic chemiluminescence image analysis system.

### siRNA Knockdown

siRNAs targeting mouse Dync1li1 and non‐targeting scramble controls were obtained from RiboBio Co., Ltd and resuspended in 10 mm Tris pH 8.0 buffer at a concentration of 20 µm. Cells were seeded in six‐well plates, grown overnight until they reached 60%–70% confluency, and then transfected with siRNA at a final concentration of 50 nm using Lipofectamine 3000. After treatment, cells were incubated for 48 h before being subjected to experiments. A full list of siRNA sequences can be found in Table S2 (Supporting Information).

### Thioflavin T Fluorometry

To quantify the amount of fibrils formation, samples were monitored by thioflavin T (ThT) fluorometry. In brief, samples were incubated with ThT (20 µm) in PBS, pH 7.4, and fluorescence was measured (λex = 450 nm, λem = 510 nm). ThT fluorescence was measured with a TECAN Spark multifunction microplate reader.

### Transmission Electron Microscopy (TEM)

SY‐SY5Y cells were treated with 20 µm cilioD or DMSO for two days. The cell precipitate was obtained by centrifugation after trypsin digestion and resuspended by adding an electron microscope fixative (G1102‐100ML, Servicebio). The cells were fixed at room temperature and protected from light for 30 min, and then transferred to 4 °C for storage. The grids were visualized with a Hitachi S‐3400N EM microscope equipment (Hitachi).

### Stereotaxic Injection of αSyn PFFs

3‐month‐old WT and 5xFAD were stereotaxically injected into one hemisphere with recombinant αSyn fibrils. A single needle insertion (coordinate: AP −2.5 mm, ML −2.0 mm, DV −1.8 mm) into the right forebrain was used to target the hippocampus. Injections were performed using a 10 µL syringe (Hamilton, NV) at a rate of 0.1 µL per min (2 µL total per site) with the needle in place for > 5 min at each target. Animals were monitored regularly following recovery from surgery and sacrificed at 7 days after injection. For histological studies, the brain was removed after transcardial perfusion with PBS and underwent overnight fix in 4% PFA (Solarbio) before being processed and embedded in an optimal cutting temperature compound (Sakura). For biochemical studies, tissues were immediately frozen after removal and stored at ‐80 °C until used.

To investigate the effect of Aβ oligomers (Aβo) on the seeding properties of αSyn PFFs, we divided 3‐month‐old mice into four groups and injected PBS, Aβo, PFFs, Aβ, and PFFs by stereotactic injection in the unilateral hippocampus. Behavioral tests were done 7 days later and sacrificed for histological studies.

To investigate the therapeutic effect of ML098 on Aβo and Lewy body comorbidities, we injected PFFs into the unilateral hippocampus of 3‐month‐old 5xFAD mice and divided the mice into two groups. Then, the control group was injected with the solvent 0.9% NaCl by intraperitoneal (i.p.) injection, and the experimental group was injected with 3.5 mg kg^−1^ of ML098 with three times a week for 6 weeks. After completion of the behavioral tests, mice were sacrificed for histological studies.

### Morris Water Maze Test

Mice were first trained for 5 days of place navigation experiments and on the sixth day for space exploration experiments. In the first 5 days, an escape platform was placed in the designated quadrant. During the training period, mice entered the pool from each of the four quadrants and were permitted to search for the platform for 60 s. The time that rats arrived at the hidden platform was regarded as escape latency. The mouse was guided onto the platform if it was not found within 60 s. On the sixth day, after the platform was removed, the time the mice swam in the target quadrant and the number of times they crossed the platform were recorded. All data in the behavioral test were analyzed by using the SMART video tracking software (Panlab).

### New Object Recognition Test

The new object recognition experiment was conducted over 2 consecutive days. After the mice acclimated to the experimental environment, we began the first day of the training experiment, observing and recording the exposure of each group of mice to two identical objects. The next day, one of the two identical objects (old object) was replaced with a different object (new object), and recorded the movement of mice in the area of new and old objects within 5 min (RWD, China).

### Statistical Analysis

All experiments were replicated at least three times. Offline data analysis was performed using ImageJ (NIH), Prism 9 (Graph Pad software), and IGOR software (Wavemetrics). When appropriate, two‐way ANOVA analyses of variance were used to test the interaction between two factors, followed by Tukey's post hoc. Comparison of groups was done by one‐way ANOVA followed by Tukey's multiple comparison test. Paired analysis was applied for repeated measurements and data originating from the same experimental subject. Outliers were removed by Grubb's tests (alpha = 0.05). All data were presented as mean ± standard error of the mean (SEM). All tests were conducted using SPSS (Statistical Package for the Social Sciences) 13.0. Values of p < 0.05 were considered to be significant.

### Study Approval

Mice were raised and maintained in a barrier facility. All animal experiments were reviewed and approved by the Institutional Animal Care and Use Committee of Tongji University, and conducted according to institutional guidelines.

## Conflict of Interest

The authors declare no conflict of interest.

## Author contributions

L.Z., Y.W., Y.L., F.Z., and G.G. contributed equally to this work. Q.W. conceived and led this study. L.Z., Y.W., G.G., and Y.L. performed cell culture experiments. L.Z., F.Z, C.L., and P.A. performed the biochemistry experiments. Y.L. and P.A. performed the RNA sequencing experiment. Y.W. and J.X. performed animal studies. L.Z., J.W., and L.G. performed histological analysis. P.A. performed computation analysis. L.Z. collected and analyzed the data. L.Z. wrote the draft. Q.W, J.Z., J.C., V.L., and Y.G. discussed and revised the manuscript. All authors read and approved the manuscript.

## Supporting information



Supporting Information

Supplemental Video 1

Supplemental Video 2

Supplemental Video 3

Supplemental Video 4

Supplemental Video 5

Supplemental Video 6

Supplemental Video 7

Supplemental Video 8

Supplemental Video 9

Supplemental Video 10

Supplemental Video 11

Supplemental Video 12

## Data Availability

The data that support the findings of this study are available from the corresponding author upon reasonable request.
