# Supplementary material for: Dynein‐Dependent Endo‐Lysosomal Degradation Drives Lewy Body Disorders Accompanied by Aβ Pathology
Source: Adv Sci (Weinh). 2025 Jul 18;12(37):e14860. doi: 10.1002/advs.202414860 (PMC12499460; doi:10.1002/advs.202414860)
Supplement: Supplementary file 1 — Supporting Information [file ADVS-12-e14860-s002.docx]

**Supplementary materials:**

**Dynein-dependent endo-lysosomal degradation drives Lewy body disorders accompanied by Aβ pathology**

Linlin Zhou ^1,#^, Yuwei Wang ^1,#^, Yu Liu ^1,#^, Feipeng Zhu ^2,#^, Ge Gao ^3,#^, Chengjie Li ^1^, Pu Ai ^1^, Jingying Xu ^4^, Junxin Wang ^3^, Long Guo ^1^, Yuting Guan ^5^, Virginia Man-Yee Lee ^6^, Jianjun Chen ^4,*^, Jialin Zheng ^3,*^, Qihui Wu ^1,*^

**Affiliations:**

^1^Shanghai Key Laboratory of Anesthesiology and Brain Functional Modulation, Clinical Research Center for Anesthesiology and Perioperative Medicine, Translational Research Institute of Brain and Brain-Like Intelligence, Shanghai Fourth People's Hospital Affiliated to Tongji University School of Medicine, State Key Laboratory of Cardiology and Medical Innovation Center, Shanghai East Hospital, School of Medicine, Tongji University, Shanghai 200092, China.

^2^State Key Laboratory for Molecular Developmental Biology, Institute of Genetics and Developmental Biology, Chinese Academy of Sciences, Beijing 100101, China.

^3^Center for Translational Neurodegeneration and Regenerative Therapy, Tongji Hospital affiliated to Tongji University School of Medicine, Shanghai Frontiers Science Center of Nanocatalytic Medicine, The Institute for Biomedical Engineering & Nano Science, School of Medicine, Tongji University, Shanghai 200092, China.

^4^Tongji University School of Medicine, 500 Zhennan Road, Shanghai, 200331, China.

^5^Shanghai Frontiers Science Center of Genome Editing and Cell Therapy, Shanghai Key Laboratory of Regulatory Biology, Institute of Biomedical Sciences and School of Life Sciences, East China Normal University, Shanghai, 200241, China.

^6^Department of Pathology and Laboratory Medicine, Institute on Aging and Center for Neurodegenerative Disease Research, Perelman School of Medicine, University of Pennsylvania, Philadelphia, PA, USA.

**^#^These authors contributed equally**.

**^*^Correspondence**: qihuiwu@tongji.edu.cn (Q.W.), jialinzheng@tongji.edu.cn (J.Z.), chenjianjun@tongji.edu.cn (J.C.)

**Supplementary figure legends:**

**
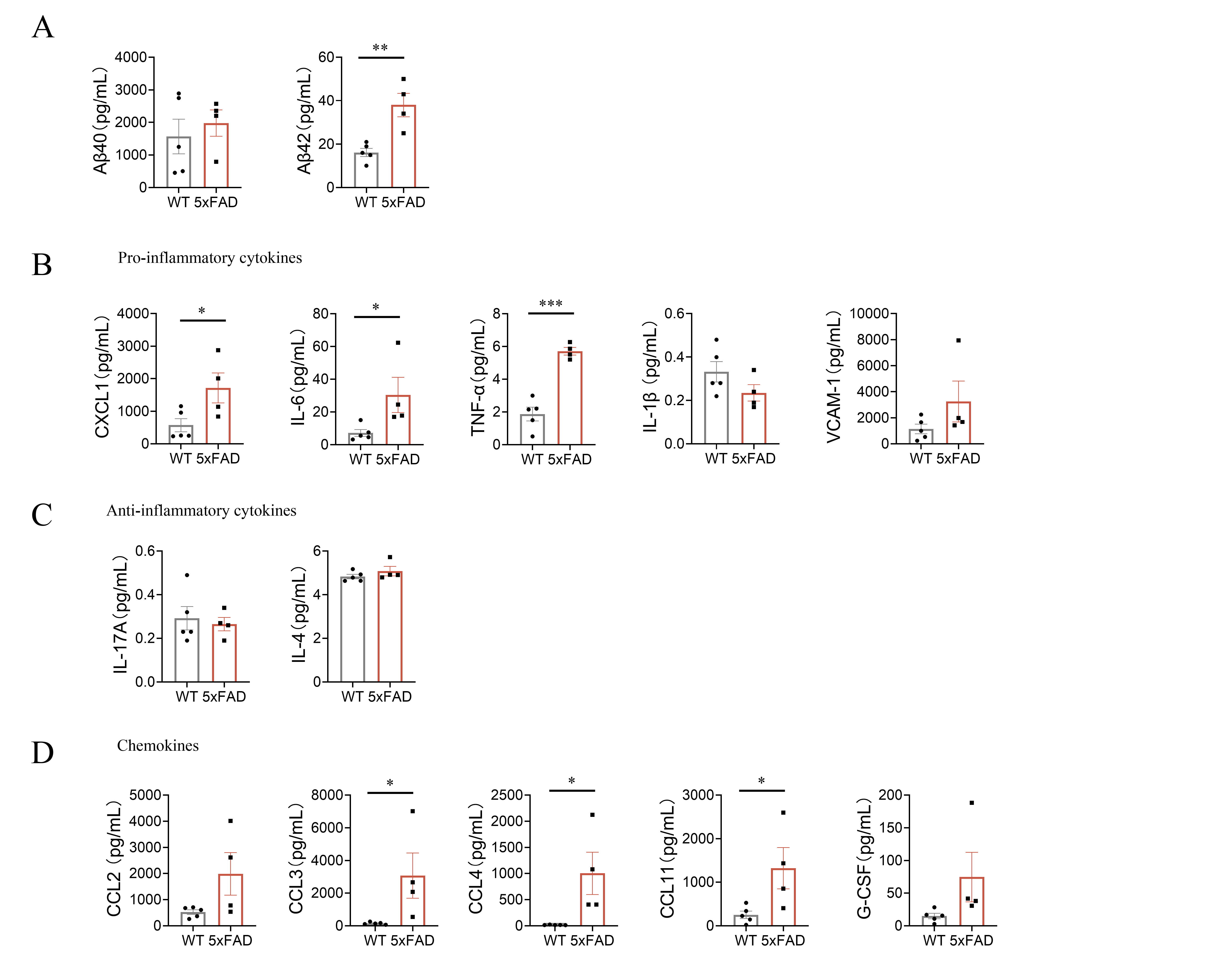
**

**Supplementary Fig 1: Characterization of slice/neuron co-culture system.**

A. Statistical quantifications of Aβ40 and Aβ42 in the culture medium collected from WT or 5xFAD slices. Brain slices were cultured for at least 7 days before been co-cultured with primary neurons or been applied in other biochemical and immunofluorescent assays. Student’s *t*-test, **p <0.01. Each dot represents a co-culture analyzed.

B. Statistical quantifications of pro-inflammatory cytokines in the culture medium collected from WT or 5xFAD slices. Student’s *t*-test, *p <0.05, ***p <0.001. Each dot represents a co-culture analyzed.

C. Statistical quantifications of anti-inflammatory cytokines in the culture medium collected from WT or 5xFAD slices. Each represents a co-culture analyzed.

D. Statistical quantifications of chemokines in the culture medium collected from WT or 5xFAD slices. Student’s *t*-test, *p <0.05. Each dot represents a co-culture analyzed.

**
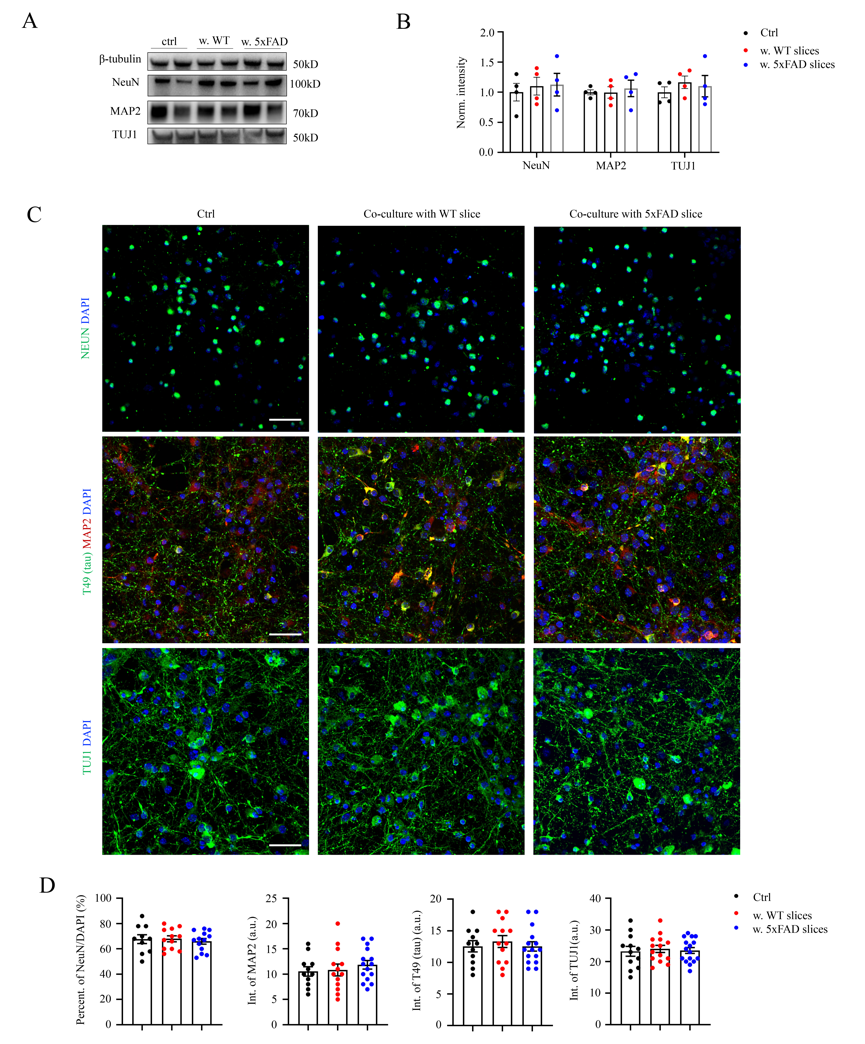
**

**Supplementary Fig 2: Co-culture with brain slices did not significantly alter the expression levels of neuronal markers.**

A and B. Western blot analysis and statistical quantifications of neuronal markers. Each dot represents a sample analyzed.

C and D. Representative confocal images and statistical quantifications of neuronal markers. Hippocampal neurons were co-cultured with brain slices for 3 days, then collected for western blot and immunofluorescent staining. Scale bar: 50 μm. Each dot represents a cell analyzed.


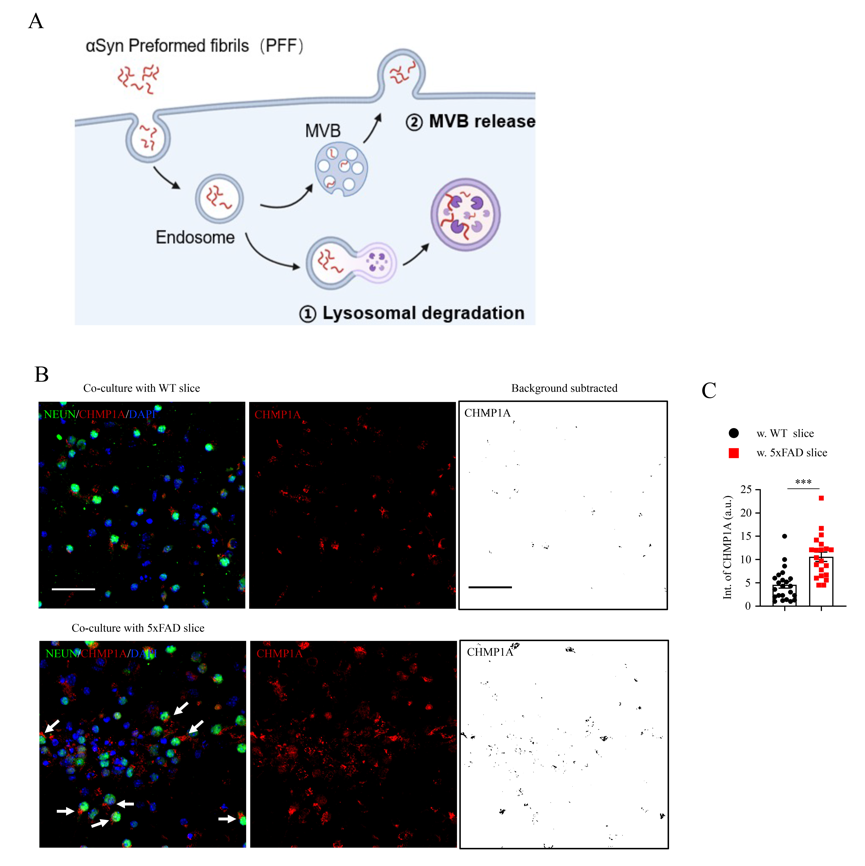


**Supplementary Fig 3: Co-culture with 5xFAD slices induced biogenesis of MVBs.**

A. Cartoon illustrates the two major pathways of αSyn PFFs after internalization into cells. After internalization, some of the αSyn PFFs were fused with endosome and were further sorted into lysosome for degradation (pathway #1); where some of the αSyn PFFs were sorted into multi-vesicular body (MVB) and were released outside of cells (pathway #2).

B. Representative confocal images of CHMP1A in neurons co-cultured with WT or 5xFAD slices. Chromatin modifying protein 1A (Chmp1A) is a member of the endosormal sorting complex required for transport (ESCRT)-III family and has been used as late endosome marker (Guan et al. 2021). Scale bar: 25 μm.

C. Statistical quantifications of CHMP1A. Student’s *t*-test, ***p <0.001. Each dot represents a ROI analyzed.

**
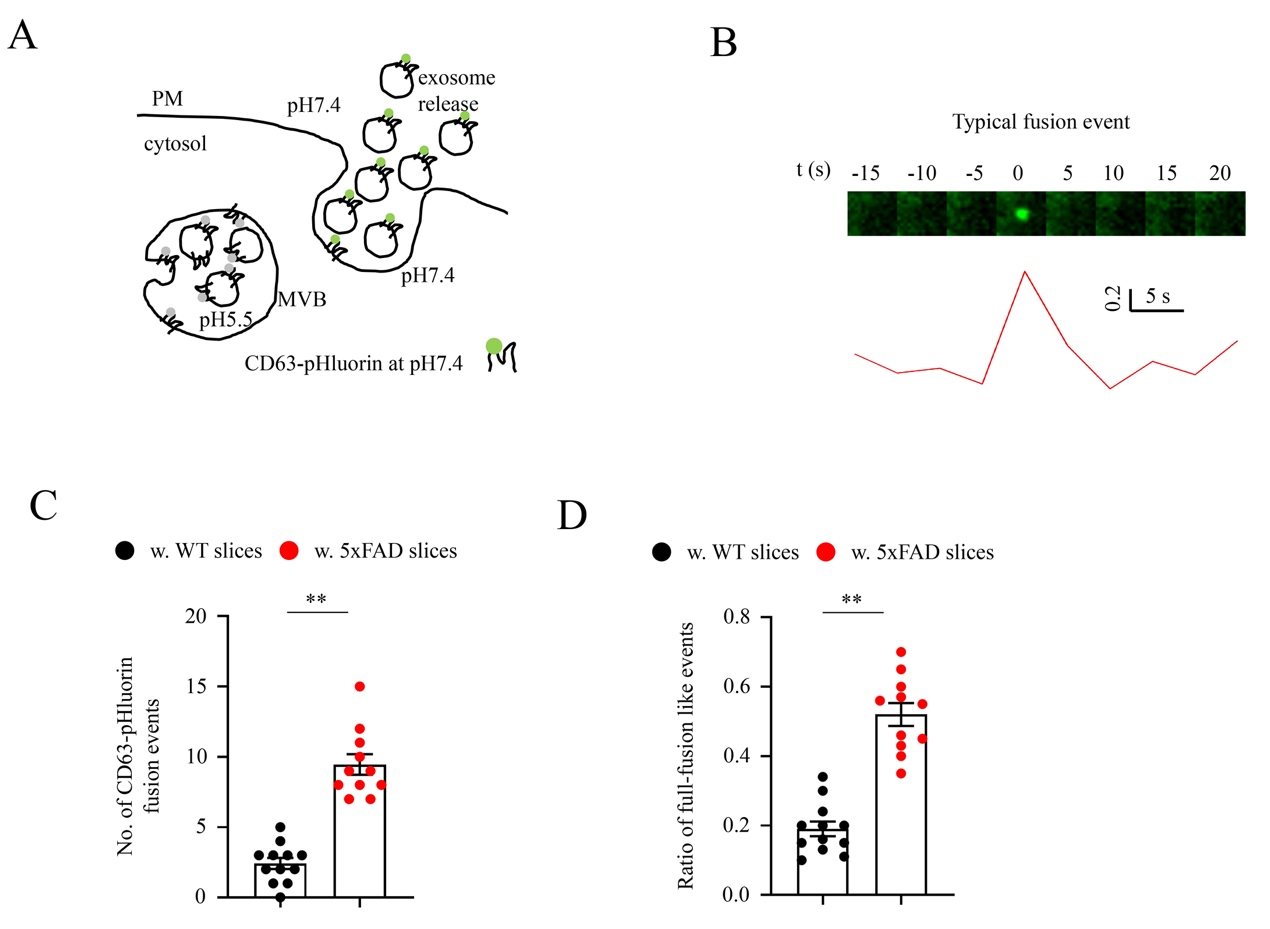
**

**Supplementary Fig 4: Confocal live imaging of CD63-pHluorin release in hippocampal neurons.**

A. Schematic illustration of CD63-pHluorin release from MVBs. The intensity of CD63-pHluorin was quenched in MVBs (pH5.5), whereas fusion of MVBs with plasma membrane resulted in increase of pH (pH7.4) and visualization of fluorescent signals.

B. Representative kymograph and fluorescent trace of a CD63-pHluorin fusion event. Scale bar: 2 μm.

C. Statistical quantifications of numbers of CD63-pHluorin fusion events in neurons co-cultured with WT or 5xFAD slices. Student’s *t*-test, **p <0.01. Each dot represents a cell analyzed.

D. Statistical quantifications of ratio of full fusion-like (FFL) events in neurons co-cultured with WT or 5xFAD slices. Following our previously defined criteria, FFL events showed a robust fluorescence increase at both the center and the annular area of CD63-pHluorin puncta, indicating the release and spread of vesicular contents (Wang et al. 2017). Student’s *t*-test, **p <0.01. Each dot represents a cell analyzed.


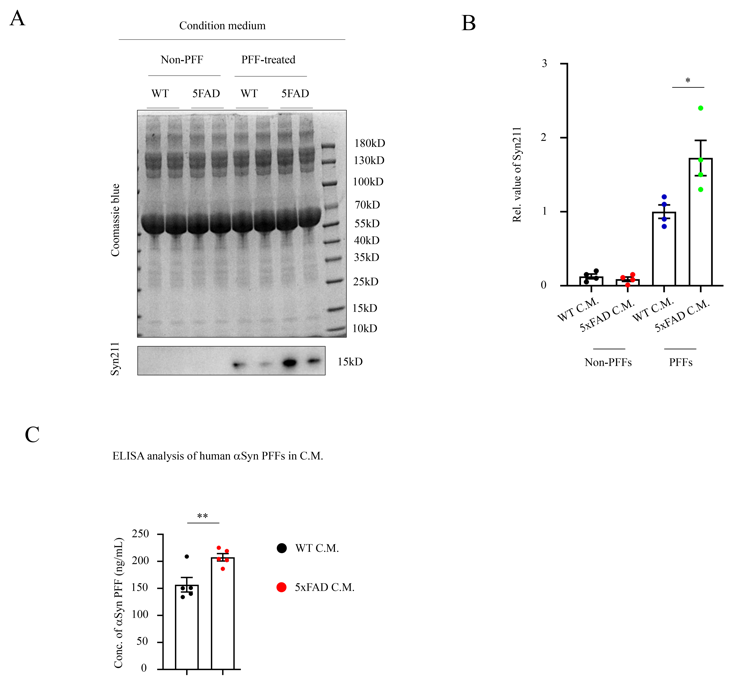


**Supplementary Fig 5: Western blot and ELISA analysis of human αSyn PFFs in conditioned medium**.

A and B. Coomassie blue staining, western blot, and quantitative analysis of human αSyn PFFs in conditioned medium. One-way ANOVA test, *p <0.05. Each dot represents a replicate of the Western blot analysis.

C. ELISA analysis of human αSyn PFFs in conditioned medium. Student’s *t*-test, **p <0.01. Each dot represents a replicate of the ELISA assays.


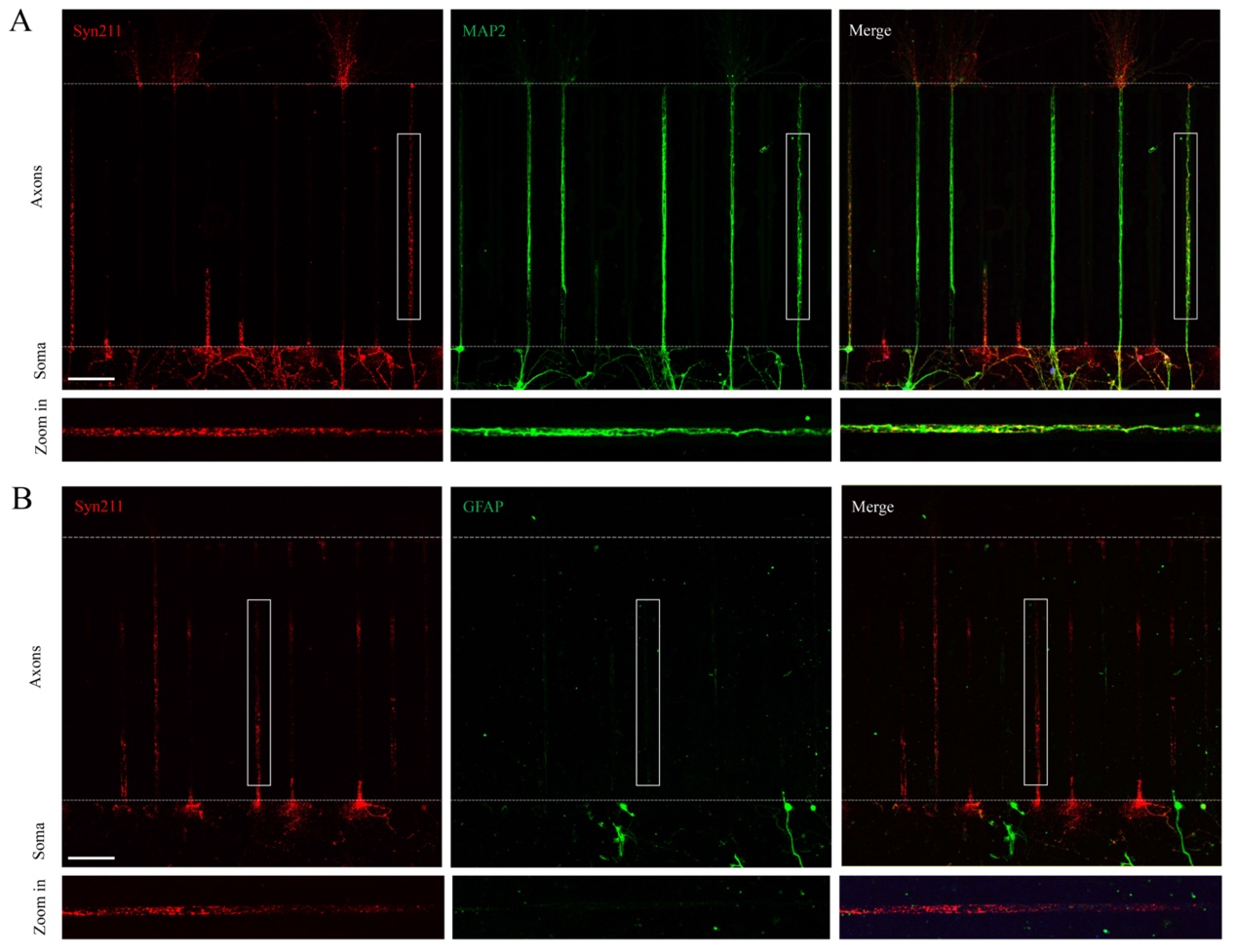


**Supplementary Fig 6: Immunofluorescent co-staining of αSyn PFF seeds with neuronal and astroglial markers.**

A and B. Immunofluorescent co-staining of Syn211 with MAP2 (A) and GFAP (B) in microfluidic chambers. Primary hippocampal neurons were cultured in the microfluidic chambers for 7 days, after which sonicated human αSyn PFFs were added into the axonal and dendritic terminals for 2 days. Immunofluorescent staining was then performed to visualize the colocalization of αSyn PFF seeds (Syn211) with neuronal (MAP2) and astroglial (GFAP) markers. In the zoomed-in panels, most αSyn PFF seeds were co-localized with MAP2 but not GFAP. Scale bar: 100 μm.


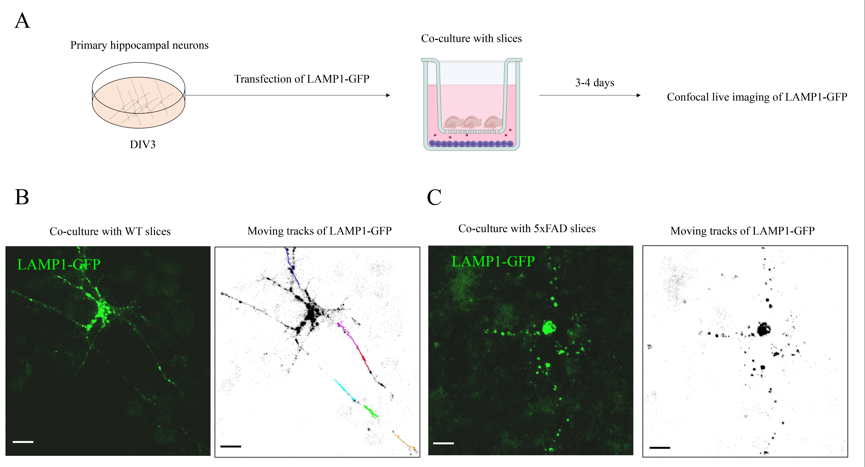


**Supplementary Fig 7: Real-time live imaging of lysosome trafficking in hippocampal neurons.**

A. Schematic illustrating the experimental protocol. Briefly, hippocampal neurons were transfected with LAMP1-GFP on DIV3 to label lysosomes. These neurons were then co-cultured with WT or 5xFAD slices for 3-4 days, followed by confocal live imaging of LAMP1-GFP.

B. Representative confocal images and movement tracks of LAMP1-GFP in hippocampal neurons co-cultured with WT or 5xFAD slices. The movement of LAMP1-GFP was manually tracked using the “MTrackJ” plugin in ImageJ software. Scale bar: 10 μm.


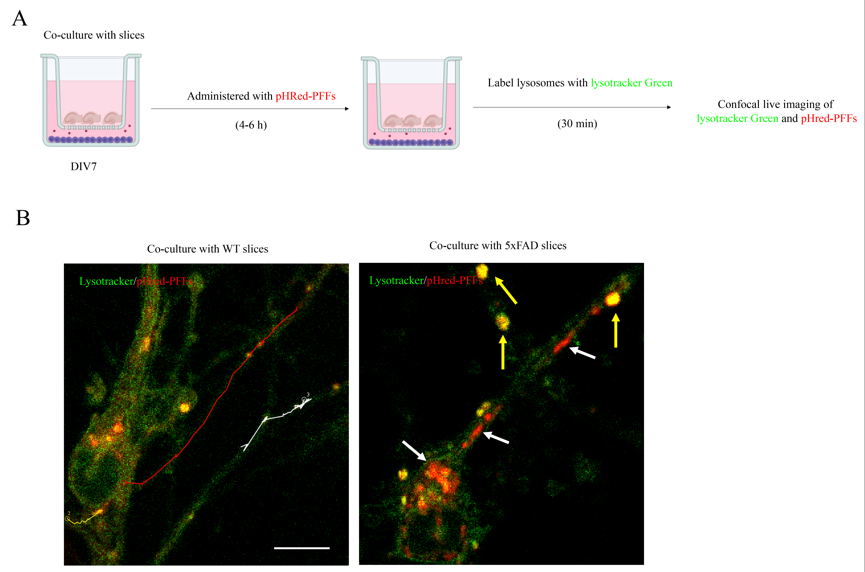


**Supplementary Fig 8: Real-time live imaging of lysosomes and αSyn PFF seed trafficking in hippocampal neurons.**

A. Schematic illustrating the experimental protocol. Briefly, hippocampal neurons were co-cultured with WT or 5xFAD slices for 7 days. The neurons were then administered αSyn pHred-PFFs for 4-6 h, followed by incubation of lysotracker green to label lysosomes. Confocal live imaging of αSyn pHred-PFFs and lysotracker green was then performed.

B. Representative confocal images and movement tracks of pHred-PFFs and lysotracker green in hippocampal neurons co-cultured with WT or 5xFAD slices. The movement of pHred-PFFs and lysotracker green was manually tracked with the “MTrackJ” plugin in ImageJ software. Yellow arrows indicate enlarged lysosome puncta in the 5xFAD condition, while white arrows indicate pHred-PFFs puncta that were not co-localized with lysotracker in the soma and axons and dendrites. Scale bar: 10 μm.


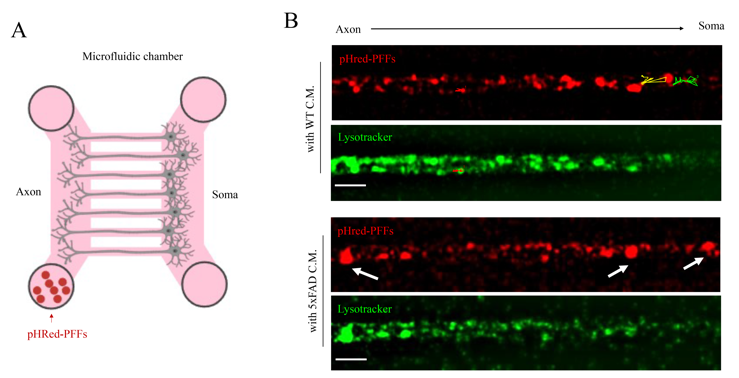


**Supplementary Fig 9: Real-time live imaging of lysosome and αSyn pHred-PFF trafficking in hippocampal neurons cultured in microfluidic chambers.**

A. Schematic illustrating the experimental protocol. Briefly, hippocampal neurons were co-cultured in microfluidic chambers with conditioned medium collected from WT or 5xFAD slices for 7 days. The neurons were then administered αSyn pHred-PFFs for 4-6 h, followed by incubation with lysotracker green to label lysosomes in the axonal and dendritic terminal chambers. Confocal live imaging of pHred-PFFs and lysotracker green was then performed.

B. Representative confocal images of αSyn pHred-PFFs and lysotracker green. White arrows indicate enlarged pHred-PFF puncta in neurons co-cultured with conditioned medium from 5xFAD slices. The movement of pHred-PFFs and lysotracker green was manually tracked with the “MTrackJ” plugin in ImageJ software. Scale bar: 5 μm.


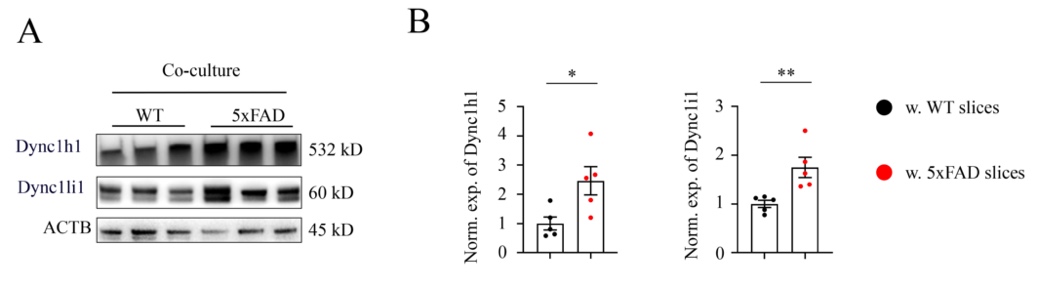


**Supplementary Fig 10: Western blot analysis of dynein complex proteins in neurons co-cultured with WT or 5xFAD slices**.

A. Western blot analysis of Dync1h1 and Dync1li1 expression levels.

B. Statistical quantifications of Dync1h1 and Dync1li1. Abbreviations: Norm. exp.: normalized expression. Student’s *t*-test, *p <0.05, **p <0.01. Each dot represents a replicate of the Western blot analysis.


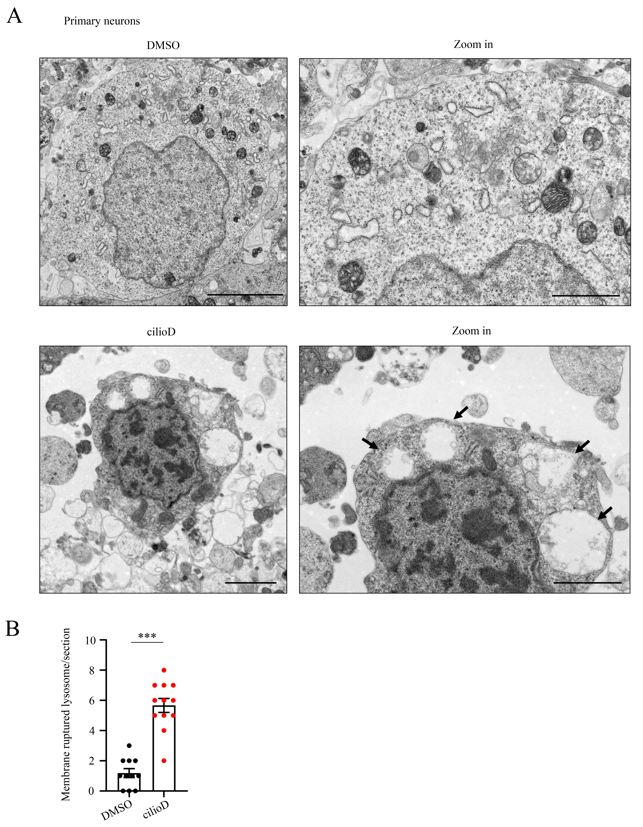


**Supplementary Fig 11: Electron microscopic images of lysosomes in DMSO- and cilioD-treated hippocampal neurons.**

A. Hippocampal neurons were treated with DMSO or cilioD overnight, then collected by centrifugation after trypsin digestion and resuspended in an electron microscopy fixative solution (G1102-100ML, Servicebio). The cells were fixed at room temperature, protected from light for 30 min, and then stored at 4°C. Grids were visualized using a Hitachi S-3400N EM microscope (Hitachi). Black arrows indicate enlarged lysosomes with ruptured membranes in cilioD-treated neurons. Scale bar: 2 μm in left panels, 5 μm in zoom-in panels.

B. Statistical quantifications of membrane ruptured lysosomes per section. Student’s *t*-test, ***p <0.001. Each dot represents a section analyzed.


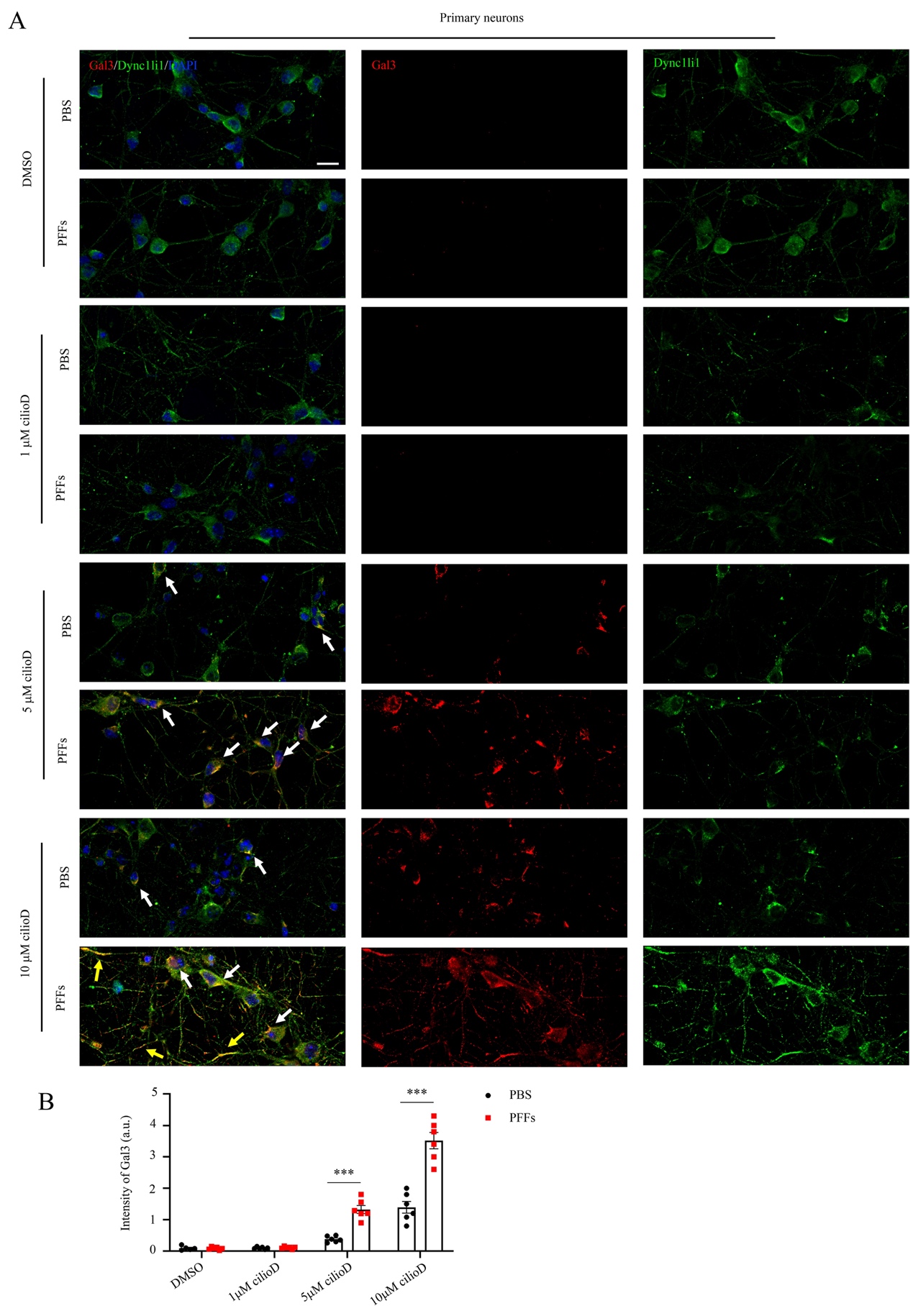


**Supplementary Fig 12: Confocal analysis of Gal3 and Dync1li1 in primary hippocampal neurons treated with cilioD and PFFs.**

A and B. Representative confocal images and statistical quantifications of Gal3 and Dync1li1 in primary hippocampal neurons under different treatments. Scale bar: 20 μm. White arrows indicate abnormal distribution of Gal3 in the soma, while yellow arrows highlight abnormal distribution of Gal3 in axons and dendrites. One-way ANOVA test, ***p <0.001. Each dot represents a region of interest (ROI) analyzed.


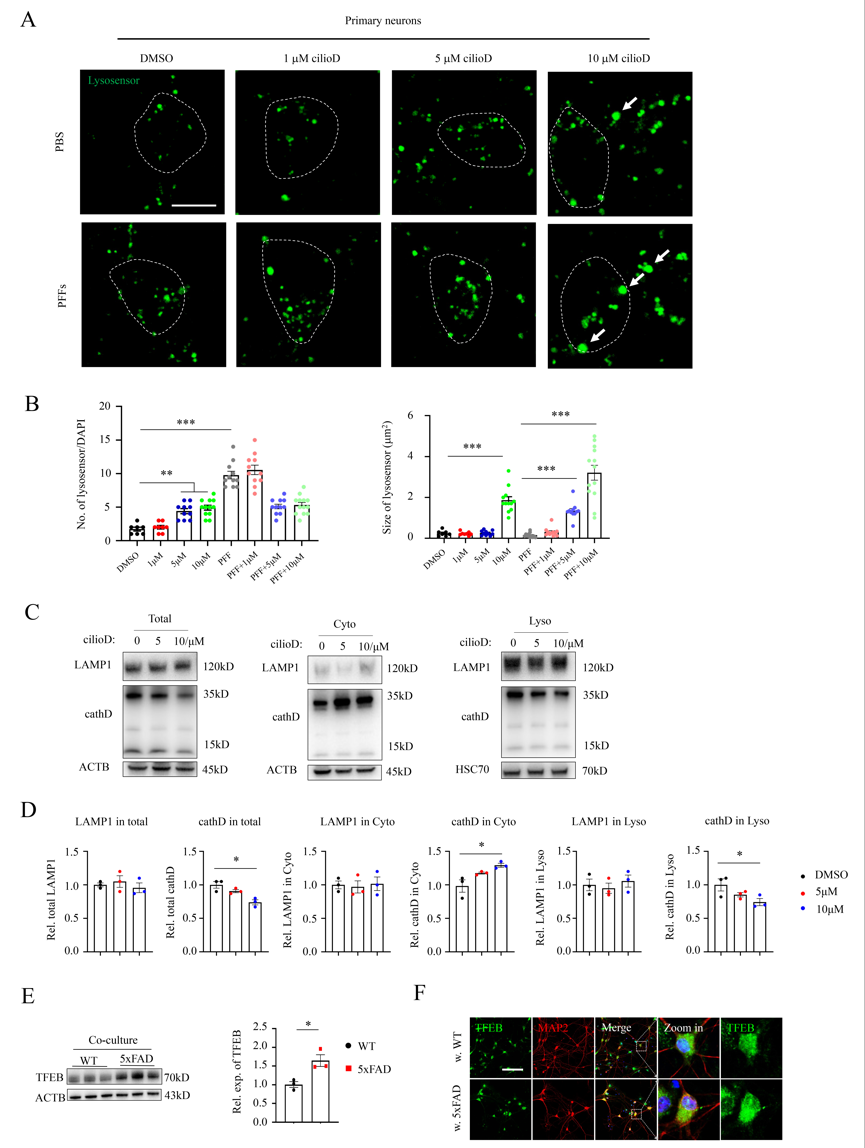


**Supplementary Fig 13: Analysis of lysosomal functions in DMSO- and cilioD-treated neurons.**

A and B. Representative confocal images and statistical quantifications of lysosensor in primary hippocampal neurons. White circles highlight the soma of neurons. Scale bar: 10 μm. One-way ANOVA test, **p <0.01, ***p <0.001. Each dot represents a cell analyzed.

C and D. Representative western blots and statistical quantifications of LAMP1 and cathD in total, cytosol (cyto) and lysosomal (lyso) fractions from primary hippocampal neurons. One-way ANOVA test, *p <0.05. Each dot represents a repeat of the western blot experiment.


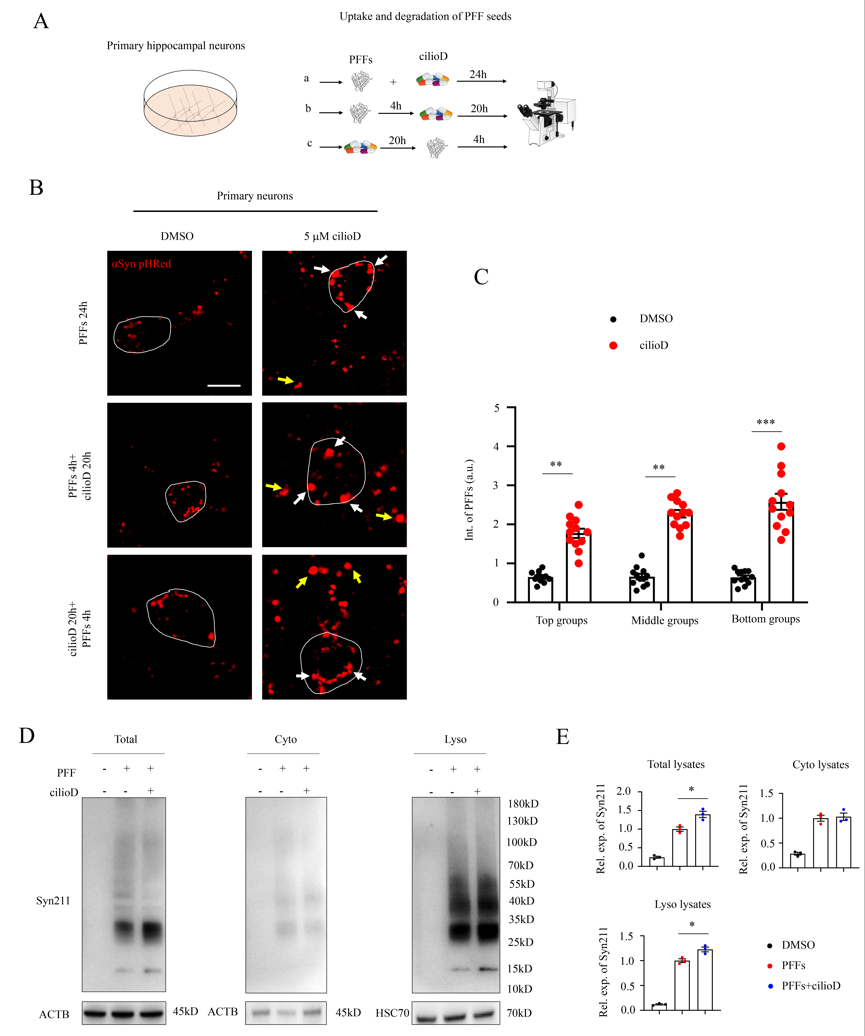


**Supplementary Fig 14: Uptake and degradation of αSyn PFFs in primary hippocampal neurons.**

A. Schematic illustration of the experimental protocol.

B and C. Representative confocal images and statistical quantifications of αSyn pHRed-PFFs in primary hippocampal neurons. Scale bar: 10 μm. White arrows indicate abnormal distribution of pHRed-PFFs in the soma, and yellow arrows highlight abnormal distribution in axons and dendrites. One-way ANOVA test, **p <0.01, ***p <0.001. Each dot represents a cell analyzed.

D. Western blot analysis of αSyn PFFs in total, cytosol, and lysosomal fractions.

E. Statistical quantifications of αSyn PFFs in total, cytosol, and lysosomal fractions. After washing the cells twice with PBS, the cell precipitate was collected by trypsin digestion and centrifugation at 1,000 g for 5 min. Reagent A was added and mixed well, and incubated at 4°C for 10 min. The mixture was homogenized using a Dounce homogenizer (30-50 strokes) and centrifuged at 2,500 g for 10 min. The supernatant was collected and centrifuged at 27,000 g for 30 min to obtain the cytosol fraction. The remaining precipitate was resuspended with PBS or Reagent B, which was used to isolate the lysosome fraction (Solarbio, EX1230). αSyn PFFs was stained with the Syn211 antibody (ab80627, Abcam) to visualize the remaining PFF seeds in different fractions. One-way ANOVA test, *p <0.05. Each dot represents a replicate of the Western blot analysis.


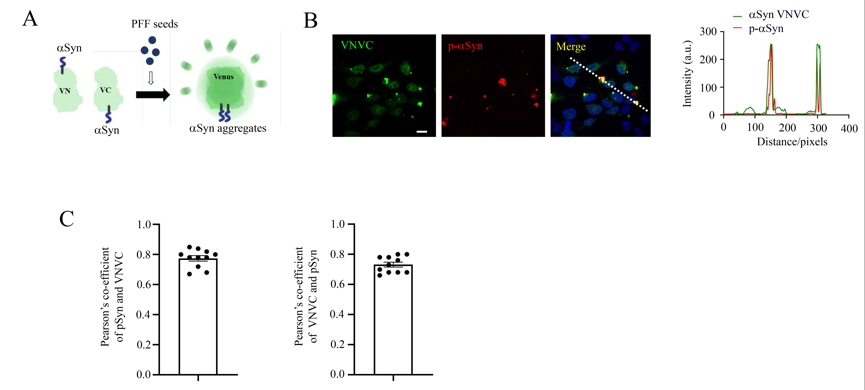


**Supplementary Fig 15: Construction of αSyn VNVC bimolecular fluorescence complementation (BiFC) probe.**

A. Cartoon illustrates the construction of αSyn VNVC bimolecular fluorescence complementation (BiFC) probe to visualize αSyn aggregation in live cells. Truncated N- or C-terminus of Venus (Addgene Plasmid #89470 and # 89471) was fused to the human αSyn and co-expressed in HEK293 cells, then selected with G418 into stable single clone, and named as αSyn VNVC.

B. Representative confocal images and plot profile distribution of the fluorescent intensity of αSyn VNVC and p-αSyn signals. VNVC puncta indicate the aggregation of αSyn. The colocalization of VNVC and p-αSyn signals indicate the success construction of the fluorescent probe. Scale bar: 10 μm.

C. Statistical quantifications of Pearson’s co-efficient of p-αSyn and VNVC (left) or VNVC and p-αSyn (right).


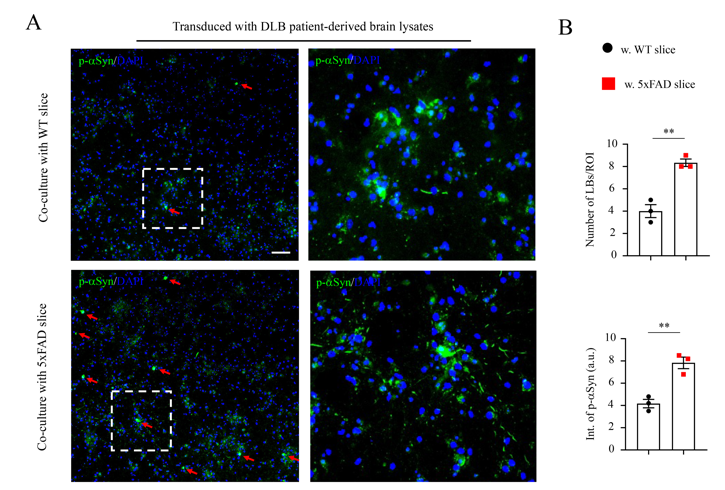


**Supplementary Fig 16: Dementia with Lewy body (DLB) patient-derived brain lysates induced p-αSyn pathologies in neurons.**

A. Representative confocal images of p-αSyn in neurons treated with dementia with Lewy body (DLB) patient-derived brain lysates. Scale bar: 50 μm.

B. Statistical quantifications of LBs (upper) and p-αSyn (lower). Student’s *t*-test, **p <0.01. Each dot represents a ROI analyzed.

**
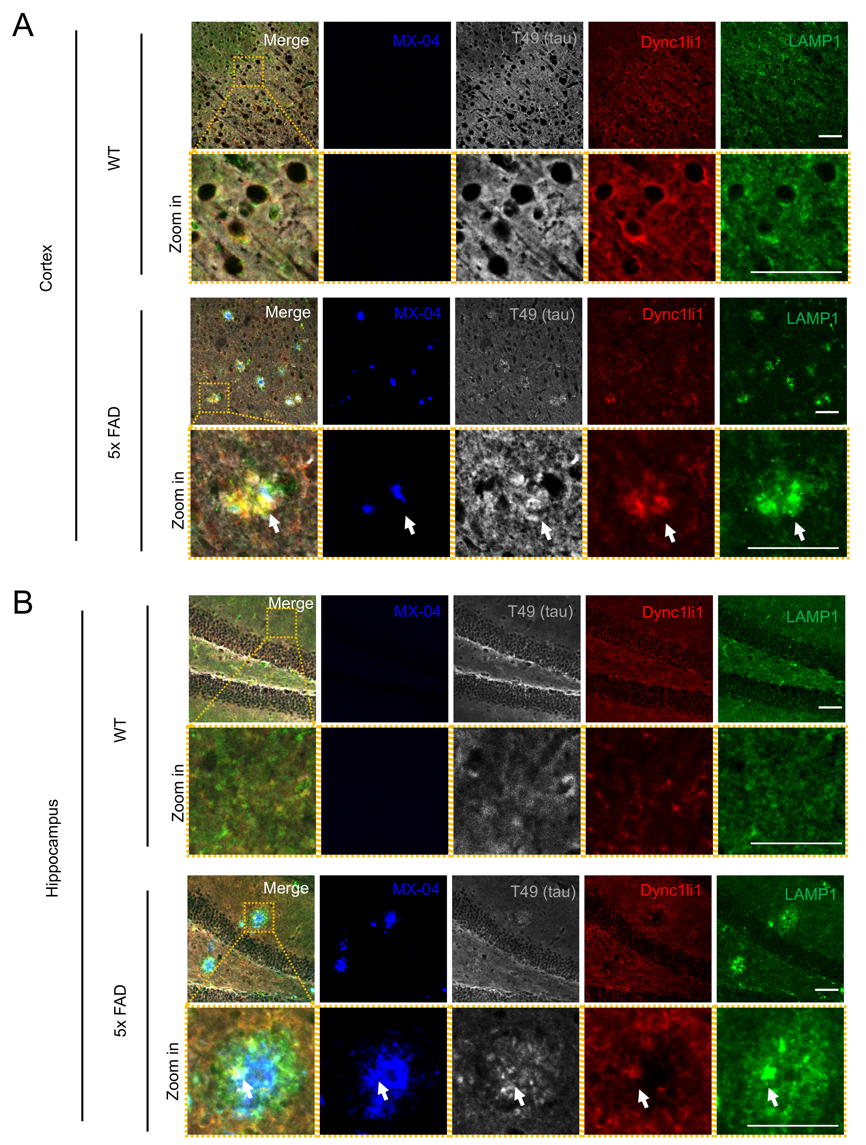
**

**Supplementary Fig 17.** Triple staining of LAMP1, Dync1li1, and tau (T49) in the cortex and hippocampus of WT and 5xFAD mice.

A and B. Immunofluorescent staining of LAMP1, Dync1li1, and neuronal marker tau (T49 antibody) in the cortex and hippocampus of WT and 5xFAD mice. Amyloid plaques were stained with MX-04 dye. White arrows indicate the co-accumulation of LAMP1, Dync1li1, and T49 around MX-04 positive plaques in the zoomed-in graphs of 5xFAD mice. Scale bar: 100 μm.

**
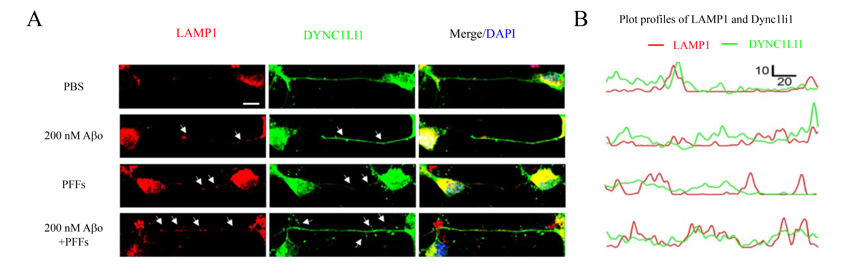
**

**Supplementary Fig 18: Aβo disrupted spatial distribution of lysosomes and dynein along axons and dendrites.**

A. Representative confocal images of LAMP1 and DYNC1LI1 in hippocampal neurons treated with αSyn PFFs or Aβo. Scale bar: 10 μm.

B. Plot profile distribution of the fluorescent intensity of LAMP1 and DYNC1LI1 along the axons and dendrites in panel (A).


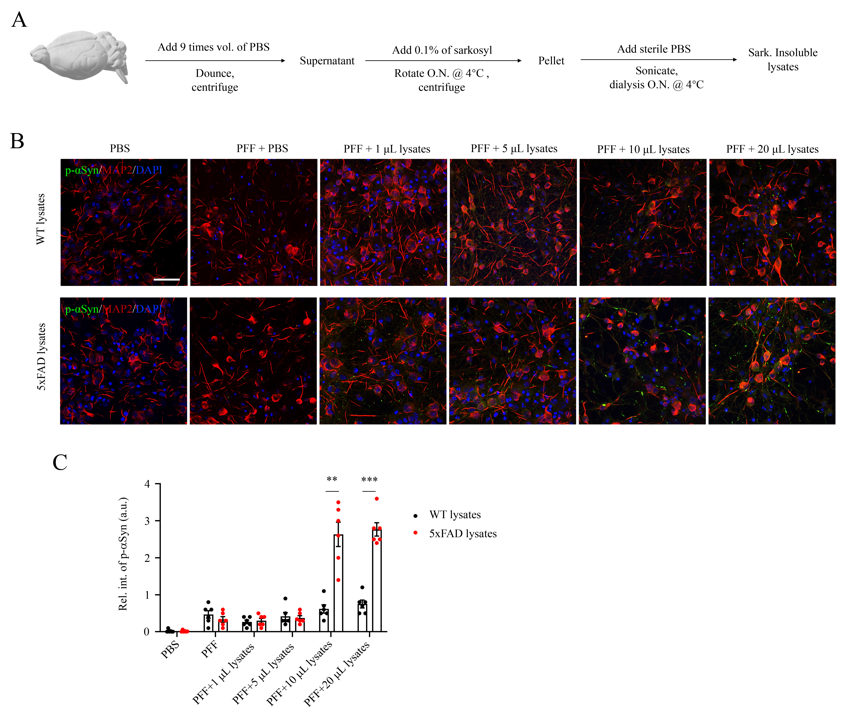


**Supplementary Fig 19: Sarkosyl-insoluble lysates extracted from 5xFAD slices induce more p-αSyn pathologies in the context of PFFs**.

A. Protocol for extracting sarkosyl-insoluble lysates from 9-month-old WT and 5xFAD mice.

B and C. Representative immunofluorescent images and statistical quantifications of p-αSyn in hippocampal neurons treated with brain lysates and αSyn PFFs. Scale bar: 100 μm. One-way ANOVA test, **p <0.01, ***p <0.001. Each dot represents a ROI analyzed.


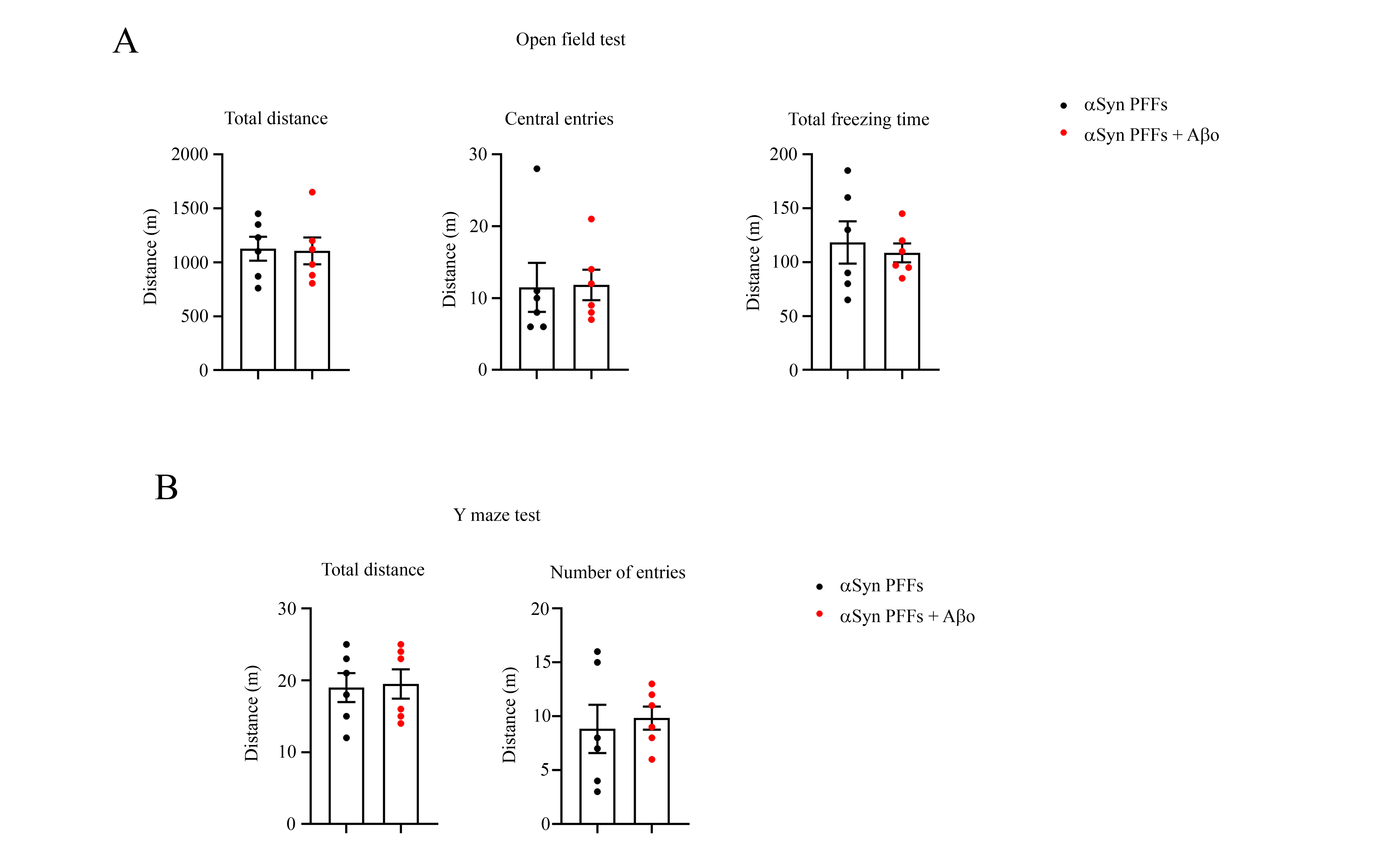


**Supplementary Fig 20: Statistical quantifications of open field and Y-maze tests in mice injected with αSyn PFFs and Aβo**.

A. Statistical quantifications of total distance, central entries, and total freezing time in the open field test. Each dot represents an animal analyzed.

B. Statistical quantifications of total distance and number of entries in the Y-maze test. Each dot represents an animal analyzed.


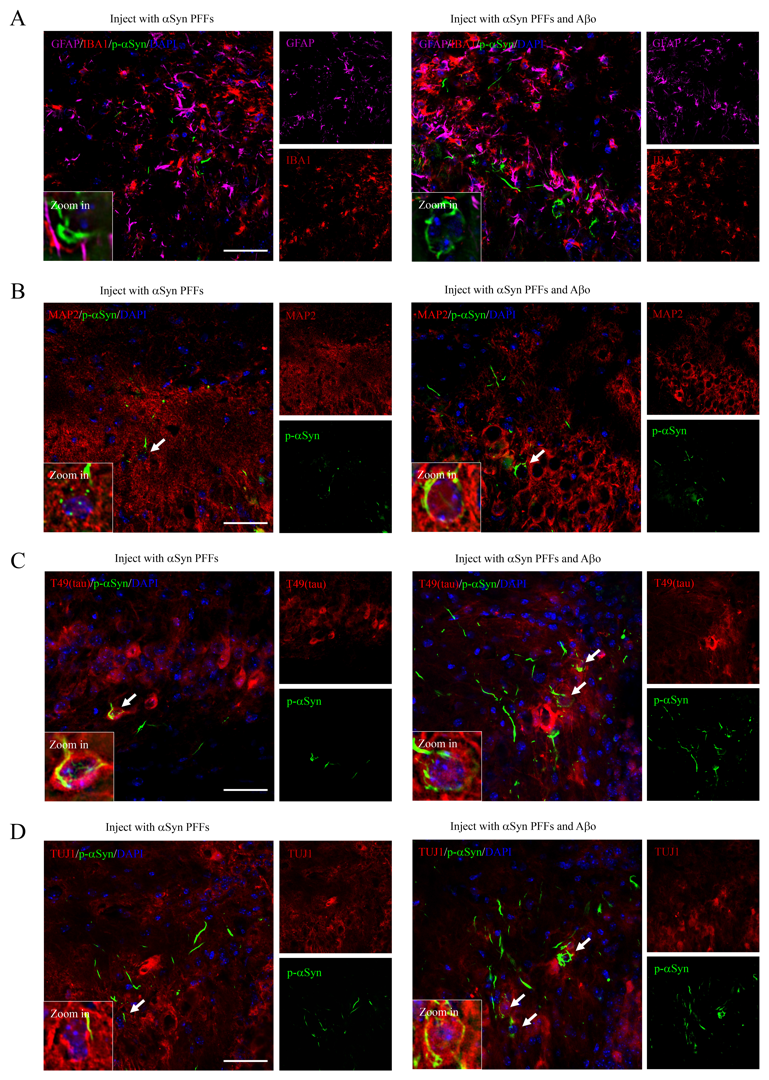


**Supplementary Fig 21: Immunofluorescent staining of p-αSyn and neuronal and glial markers in mice injected with αSyn PFFs and Aβo**.

A. Representative immunofluorescent images showing p-αSyn and GFAP/IBA1 in mice injected with αSyn PFFs and Aβo. Scale bar: 100 μm.

B-D. Representative immunofluorescent images showing p-αSyn and neuronal markers: MAP2 (B), tau (C), and TUJ1 (D) in mice injected with αSyn PFFs and Aβo. White arrows indicate co-localization of p-αSyn and neuronal markers. Scale bar: 100 μm.


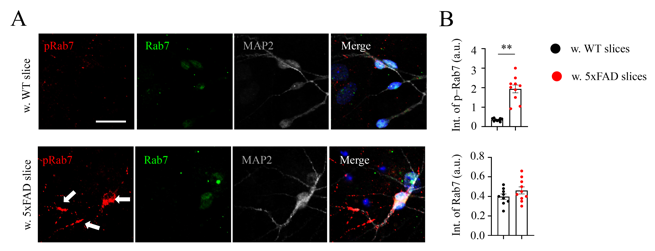


**Supplementary Fig 22: Abnormal accumulation of pRab7 in neurons co-cultured with 5xFAD slices.**

A. Representative confocal images of pRab7, Rab7 and MAP2 in hippocampal neurons co-cultured with WT or 5xFAD slices. Scale bar: 50 μm.

B. Statistical quantifications of pRab7 and Rab7 in neurons co-cultured with WT or 5xFAD slices. Student’s *t*-test, **p <0.01. Each dot represents a cell analyzed.


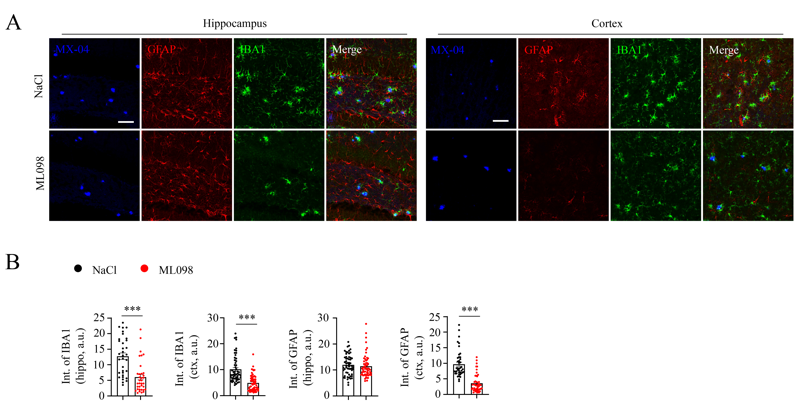


**Supplementary Fig 23: Immunofluorescent analysis of IBA1 and GFAP in hippocampus and cortex.**

A. Representative confocal images of IBA1 and GFAP in hippocampus (left) and cortex (right) with NaCl or ML098 treatment. Scale bar: 100 μm.

B. Statistical quantifications of IBA1 and GFAP. Student’s *t*-test, ***p <0.001. Each dot represents a cell analyzed.

**
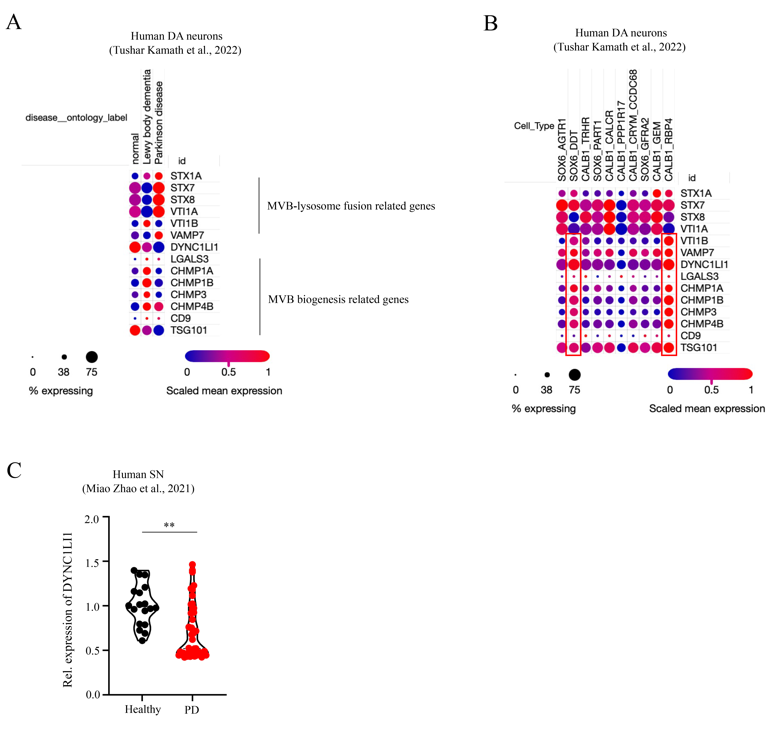
**

**Supplementary Fig 24: Bioinformatic analysis of MVB-lysosome fusion- and MVB biogenesis-related genes in publicly available single-cell RNA sequencing datasets.**

A. Dot plot of MVB-lysosome fusion- and MVB biogenesis-related genes in human dopamine neuron (DA) of normal, Lewy body dementia and PD patients from Tushar Kamath et al., 2022 dataset (Kamath et al. 2022).

B. Dot plot of MVB-lysosome fusion- and MVB biogenesis-related genes within human dopamine neuron (DA) subcluters from Tushar Kamath et al., 2022 dataset (Kamath et al. 2022).

C. Re-analysis of relative expression of DYNC1LI1 gene in human substantia nigra (SN) tissues from Miao Zhao et al., 2021 dataset (Zhao et al. 2021).

**List of Supplementary videos:**

Supplementary Video 1: Live imaging of neurons transfected with CD63-pHluorin plasmids with WT slice co-cultures.

Supplementary Video 2: Live imaging of neurons transfected with CD63-pHluorin plasmids with 5xFAD slice co-cultures.

Supplementary Video 3: Live imaging of neurons transfected with LAMP1-GFP plasmids with WT slice co-cultures.

Supplementary Video 4: Live imaging of neurons transfected with LAMP1-GFP plasmids with 5xFAD slice co-cultures.

Supplementary Video 5: Live imaging of neurons labeled with lysotracker and pHRed-PFFs with WT slice co-cultures.

Supplementary Video 6: Live imaging of neurons labeled with lysotracker and pHRed-PFFs with 5xFAD slice co-cultures.

Supplementary Video 7: Live imaging of neurons in microfluidic chambers labeled with pHRed-PFFs with WT slice conditioned medium.

Supplementary Video 8: Live imaging of neurons in microfluidic chambers labeled with lysotracker with WT slice conditioned medium.

Supplementary Video 9: Live imaging of neurons in microfluidic chambers labeled with lysotracker and pHRed-PFFs with WT slice conditioned medium.

Supplementary Video 10: Live imaging of neurons in microfluidic chambers labeled with pHRed-PFFs with 5xFAD slice conditioned medium.

Supplementary Video 11: Live imaging of neurons in microfluidic chambers labeled with lysotracker with 5xFAD slice conditioned medium.

Supplementary Video 12: Live imaging of neurons in microfluidic chambers labeled with lysotracker and pHRed-PFFs with 5xFAD slice conditioned medium.

**Supplementary Table 1. Reagents and software used in this study.**

| **REAGENT Or RESOURCE** | **SOURCE** | **IDENTIFIER** |
| --- | --- | --- |
| **Antibodies and Dyes** | | |
| Purified anti-α-Synuclein Antibody | Biolegend | 825701 |
| Anti-MAP2 Antibody | Rockland | 200-901-D68 |
| Anti-Alpha-synuclein [syn211] | Abcam | ab80627 |
| Anti-Alpha-synuclein (phospho S129) [EP1536Y] | Abcam | ab51253 |
| β-Actin Rabbit mAb (High Dilution) | Abclonal | AC026 |
| Anti-LAMP1 | Abcam | ab24170 |
| CD107a / LAMP1 Monoclonal antibody | proteintech | 67300-1-Ig |
| Galectin 3 Monoclonal Antibody | Invitrogen | 2362881 |
| Cathepsin D Rabbit Monoclonal Antibody | Beyotime | AF1645 |
| DYNC1LI1 Polyclonal antibody | proteintech | 25326-1-AP |
| Anti-GAPDH | Abmart | P60037 |
| Anti-LRRK1 | Abmart | MU126014 |
| Anti-RAB7[Rab7-117] | Abcam | ab50533 |
| Anti-RAB7 | CST | 9367 |
| Anti-RAB7 (phospho S72) antibody [MJF-R38-1] | Abcam | ab302494 |
| LysoSensor Green DND-189 | YEASEN | 40767ES50 |
| LysoTracker Red DND-99 | YEASEN | 40739ES50 |
| LysoTracker Green DND-26 | MAOKANG | L7256 |
| Methoxy-X04 | MCE | HY-103240 |
| Hoechst | MCE | 33258 |
| Goat Anti-Rabbit IgG-HRP | Abmart | M21002 |
| Goat Anti-Mouse IgG-HRP | Abmart | M21001 |
| Goat anti-Rabbit IgG (H+L) Cross-Adsorbed Secondary Antibody, Alexa Fluor™ 488 | Invitrogen | A-11008 |
| Goat anti-Mouse IgG (H+L) Cross-Adsorbed Secondary Antibody, Alexa Fluor™ 488 | Invitrogen | A-11001 |
| Goat anti-Rabbit IgG (H+L) Cross-Adsorbed Secondary Antibody, Alexa Fluor™ 568 | Invitrogen | A-11011 |
| Goat anti-Mouse IgG (H+L) Cross-Adsorbed Secondary Antibody, Alexa Fluor™ 568 | Invitrogen | A-11004 |
| Goat anti-Chicken IgY (H+L) Secondary Antibody, Alexa Fluor™ 647 | Invitrogen | A-21449 |
| **Chemicals, Peptides, and Recombinant Proteins** | | |
| Thioflavin T | Abcam | ab120751 |
| Opti-MEM | Gibco | 31985070 |
| NEUROBASAL | Gibco | 21103049 |
| NEUROBASAL-A | Gibco | 10888022 |
| FBS | Gibco | 10270106 |
| Horse serum | Gibco | 26050088 |
| B-27 | Thermo | 0080085SA |
| 100X Glutamax | Thermo | 35050061 |
| DMEM | Procell | PM150210 |
| PBS | Procell | PB180327 |
| PENICILLIN STREPTOMYCIN SOL | Gibco | 15070063 |
| L-Cysteine | Biofroxx | 1206GR025 |
| Thioflavin S | SANTACRUZ | sc-391005 |
| HEPES sodium salt solution (1M) | SANTACRUZ | sc-300789 |
| Hank's Balanced Salt Solution (HBSS) | Cytiva | SH30588.01 |
| Clodronate disodium tetrahydrate | TargetMol | T3280 |
| Trans Liposomal Transfection Reagent | Yeasen | 40802ES02 |
| Recombinant Human BDNF | Peprotech | 450-02 |
| DNase Vial (D2) | Worthington | LK003172 |
| Papain, Suspension | Worthington | LS003126 |
| 0.5 M EDTA, pH8.0 | Beyotime | ST066 |
| D-Lysine homopolymer hydrobromide | Sigma-Aldrich | P0899-100MG |
| Lentivirus Concentration Solution | Yeasen | 41101ES50 |
| Paraformaldehyde, 4％ | Solarbio | P1110 |
| Triton X-100 | Solarbio | T8200 |
| Bovine Serum Albumin | Sigma-Aldrich | B2064-100G |
| pHrodo™ Red Microscale Labeling Kit | Thermo | P35363 |
| M-PER Mammalian Protein Extraction Reagent | Thermo | 78501 |
| T-PER Tissue Protein Extraction Reagent | Thermo | 78510 |
| Fast preparation kits of PAGE gel | Epizyme | PG112/PG113 |
| FuturePAGE 4-12% 12/15 wells | ACE | ET12420/ET15420 |
| SDS-PAGE loading buffer(5X) | Beyotime | P0015L |
| Phosphatase Inhibitor Cocktail (2 Tubes, 100X) | Apexbio | K1015 |
| Protease Inhibitor Cocktail (EDTA-Free, 100 X in DMSO) | Apexbio | K1007 |
| Ciliobrevin D | MCE | HY-122632 |
| ML-098 (Rab7 agonist) | MCE | HY-19800 |
| Universal Antibody Diluent | NCM biotech | WB500D |
| NaCl | Sigma | S9625-500G |
| KCl | Sigma | P5405-250G |
| NaH_2_PO_4_·H_2_O | Sigma | S9638-25G |
| MgCl_2_·6H_2_O | Sigma | M2393-100G |
| CaCl_2_·2H_2_O | Sigma | C3306-100G |
| Glucose | Sigma | D9434-250G |
| NaHCO_3_ | Sigma | S5761-500G |
| **Experimental Models: Cell Lines** | | |
| HEK293T cells | this paper |  |
| α-Synuclein VN/VC cell line | this paper |  |
| human α-Synuclein A53T HEK293T stable cell | this paper |  |
| **Plasmids** | | |
| Syn A53T pLVX tight puro | Gift from Prof. Virginia Lee (UPENN) |  |
| VN-alpha Synuclein | addgene | 89470 |
| alpha Synuclein-VC | addgene | 89471 |
| CD63-pHluorin | this paper |  |
| CD63-pEGFP C2 | addgene | 62964 |
| **Software and Algorithms** | | |
| GraphPad Prism | GraphPad | https://www.graphpad.com |
| ImageJ (Fiji version) | NIH | https://imagej.net/Fiji/Downloads |
| Adobe Illustrator | adobe | https://www.adobe.com/products/illustrator/ |
| Biorender |  | https://www.biorender.com/ |

**Supplementary Table 2. Sequences of Dync1li1-targeted siRNAs and ASOs**

| **DYNC1LI1** | **SEQUENCE** | **SOURCE** |
| --- | --- | --- |
| siRNA 1 | GTGGGATAATGATAAGAAA | RIBOBIO |
| siRNA 2 | GTGGGATAATGATAAGAAA | RIBOBIO |
| siRNA 3 | GCTGGTTGTTGACATGTCA | RIBOBIO |
| ASO1 | FAM-ACAGAACTTGCGGATG | TSINGKE |
| ASO2 | FAM-GAAGTCTCTAATCAAC | TSINGKE |
| ASO3 | FAM-GTCGTGCACATTTAAG | TSINGKE |
| ASO scramble | FAM-CACAGCGTATAGAGGT | TSINGKE |

**References:**

Guan, Y., X. Liang, Z. Ma *et al.* 2021. "A Single Genetic Locus Controls Both Expression of Dpep1/Chmp1a and Kidney Disease Development Via Ferroptosis." *Nat Commun* 12 (1):5078. <https://doi.org/10.1038/s41467-021-25377-x>.

Kamath, T., A. Abdulraouf, S. J. Burris *et al.* 2022. "Single-Cell Genomic Profiling of Human Dopamine Neurons Identifies a Population That Selectively Degenerates in Parkinson's Disease." *Nat Neurosci* 25 (5):588-95. <https://doi.org/10.1038/s41593-022-01061-1>.

Wang, Y., Q. Wu, M. Hu *et al.* 2017. "Ligand- and Voltage-Gated Ca(2+) Channels Differentially Regulate the Mode of Vesicular Neuropeptide Release in Mammalian Sensory Neurons." *Sci Signal* 10 (484)<https://doi.org/10.1126/scisignal.aal1683>.

Zhao, M., B. Wang, C. Zhang *et al.* 2021. "The Dj1-Nrf2-Sting Axis Mediates the Neuroprotective Effects of Withaferin a in Parkinson's Disease." *Cell Death Differ* 28 (8):2517-35. <https://doi.org/10.1038/s41418-021-00767-2>.
